# Multidrug-resistant *Neisseria gonorrhoeae* infection in heterosexual men with reduced susceptibility to ceftriaxone, first report in Thailand

**DOI:** 10.1038/s41598-021-00675-y

**Published:** 2021-11-04

**Authors:** Naris Kueakulpattana, Dhammika Leshan Wannigama, Sirirat Luk-in, Parichart Hongsing, Cameron Hurst, Vishnu Nayak Badavath, Piroon Jenjaroenpun, Thidathip Wongsurawat, Nipat Teeratakulpisan, Stephen J. Kerr, Shuichi Abe, Phatthranit Phattharapornjaroen, Aye Mya Sithu Shein, Thammakorn Saethang, Naphat Chantaravisoot, Mohan Amarasiri, Paul G. Higgins, Tanittha Chatsuwan

**Affiliations:** 1 Department of Microbiology, Faculty of Medicine, Chulalongkorn University, King Chulalongkorn Memorial Hospital, Thai Red Cross Society, Bangkok, King Thailand; 2 School of Medicine, Faculty of Health and Medical Sciences, The University of Western Australia, Nedlands, WA Australia; 3 Antimicrobial Resistance and Stewardship Research Unit, Faculty of Medicine, Chulalongkorn University, Bangkok, Thailand; 4 Department of Clinical Microbiology and Applied Technology, Faculty of Medical Technology, Mahidol University, Bangkok, Thailand; 5 Mae Fah Luang University Hospital, Mae Fah Luang University, Chiang Rai, 57100 Thailand; 6 School of Integrative Medicine, Mae Fah Luang University, Chiang Rai, 57100 Thailand; 7 Department of Statistic, QIMR Berghofer Medical Research Institute, Brisbane, QLD Australia; 8 Institute for Drug Research, The Hebrew University, Jerusalem, 9112001 Israel; 9 Chitkara College of Pharmacy, Chitkara University, Punjab, 140401 India; 10 Division of Bioinformatics and Data Management for Research, Department of Research and Development, Faculty of Medicine, Siriraj Hospital, Mahidol University, Bangkok, 10700 Thailand; 11 The Thai Red Cross AIDS Research Centre, Bangkok, Thailand; 12 HIV-NAT, Thai Red Cross AIDS Research Centre, Bangkok, Thailand; 13 Center of Excellence in Biostatistics, Faculty of Medicine, Chulalongkorn University, Bangkok, Thailand; 14 Department of Infectious Diseases and Infection Control, Yamagata Prefectural Central Hospital, Yamagata, Japan; 15 Department of Emergency Medicine, Center of Excellence, Faculty of Medicine Ramathibodi Hospital, Mahidol University, Bangkok, Thailand; 16 Department of Computer Science, Faculty of Science, Kasetsart University, Bangkok, Thailand; 17 Office of Research Affairs, Faculty of Medicine, Chulalongkorn University, Bangkok, Thailand; 18 Department of Biochemistry, Faculty of Medicine, Chulalongkorn University, Bangkok, Thailand; 19 Laboratory of Environmental HygieneDepartment of Health Science, School of Allied Health Sciences, Kitasato University, Sagamihara-Minami, KitasatoKanagawa, 252-0373 Japan; 20 Institute for Medical Microbiology, Immunology and Hygiene, Faculty of Medicine and University Hospital Cologne, University of Cologne, Cologne, Germany; 21 German Centre for Infection Research, Partner Site Bonn-Cologne, Cologne, Germany

**Keywords:** Microbiology, Molecular biology, Molecular medicine, Infectious diseases, Urogenital diseases, Infection

## Abstract

The global rapid emergence of azithromycin/ceftriaxone resistant *Neisseria gonorrhoeae* threatens current recommend azithromycin/ceftriaxone dual therapy for gonorrhea to ensure effective treatment. Here, we identified the first two *N. gonorrhoeae* isolates with decreased ceftriaxone susceptibility in Thailand. Among 134 *N. gonorrhoeae* isolates collected from Thai Red Cross Anonymous Clinic, Bangkok, two isolates (NG-083 and NG-091) from urethral swab in male heterosexual patients had reduced susceptibility to ceftriaxone (MICs of 0.125 mg/L). Both were multidrug resistant and strong biofilm producers with ceftriaxone tolerance (MBEC > 128 mg/L). NG-083 and NG-091 remained susceptible to azithromycin (MIC of 1 mg/L and 0.5 mg/L, respectively). Reduced susceptibility to ceftriaxone was associated with alterations in PBP2, PBP1, PorB, MtrR, and *mtrR* promoter region. NG-083 belonged to sequence type (ST) 7235 and NG-091 has new allele number of *tbpB* with new ST. Molecular docking revealed ceftriaxone weakly occupied the active site of mosaic XXXIV penicillin-binding protein 2 variant in both isolates. Molecular epidemiology results revealed that both isolates display similarities with isolates from UK, USA, and The Netherlands. These first two genetically related gonococcal isolates with decreased ceftriaxone susceptibility heralds the threat of treatment failure in Thailand, and importance of careful surveillance.

## Introduction

*Neisseria gonorrhoeae* is the second most common bacterial sexually transmitted infection and results in substantial morbidity^1^. The World Health Organization (WHO) estimates that in 2016, 86.9 million gonococcal cases occurred among adolescents and adults worldwide^2^. Most causes are asymptomatic and predominantly in male and female under the age of 25^1,3^. Complication of untreated gonorrhoea can be leads to major morbidities such as pelvic inflammatory disease, ectopic pregnancies, and infertility in women^1^. It also increases the risk of human immunodeficiency virus (HIV) transmission and acquisition among man who having sex with man and heterosexual individuals^1,3^. The youngrace or ethnicity, urban residence, multiple, sequential, or concurrent sexual relationships, inconsistent use of condoms, and frequent use of alcohol, illicit substances^4^ and antibiotic resistance are related to increased transmission of gonorrhoea infections^3^.

In *N. gonorrhoeae*, resistance to penicillin, tetracycline, and fluoroquinolones has successively developed, limiting the therapeutic use of these antibiotics^1,5,6^. Currently, the injectable third generation cephalosporin (ceftriaxone) in combination with a macrolide (azithromycin) are recommended by the World Health Organization (WHO) as the antibiotics of choice to treat most gonococcal infections^7^ while the Centre for Disease Control and Prevention (CDC) recommend the injectable ceftriaxone^2^. However, increasing reports of *N. gonorrhoeae* infections with azithromycin resistance, and ceftriaxone reduced susceptibility or resistantance, threatens this regimen^1,8–10^. *N. gonorrhoeae* strains with resistance or reduced susceptibility to ceftriaxone have now been reported in several other countries^1,11–19^. In *N. gonorrhoeae*, mutations within the penicillin-binding protein either PBP1 or PBP2 lead to elevated minimum inhibitory concentrations (MICs) via decreased affinity to β-lactams^1,14,20–22^. An alternative mechanism of β-lactam resistance involves overexpression of the MtrCDE efflux pump or reduced outer membrane permeability by mutations of the *mtrR* and *por* genes^1,21,22^. Mosaic-like structures in the *penA* gene which encodes PBP2 are known to be associated with high-level resistance to penicillin and reduced susceptibility to ceftriaxone, cefipime and other cephems^1,11,14–16^. Additionally, PBP2 the amino acid substitutions G542S, P551S, and P551L in nonmosaic *penA* alleles have been reported in isolates with reduced susceptibility to extended spectrum cephalosporins^1,11,14–16^.

No confirmed reports of gonorrhoea resistance or reduced susceptibility to ceftriaxone have been documented in Thailand to date. In current gonococcal treatment in Thailand, azithromycin is added to ceftriaxone in dual therapy as a method of limiting the selection of ceftriaxone resistant mutants. Thailand is a major risk area for gonorrhea because it is a key destination for the sex tourism industry, and where antibiotic resistant gonorrhea can appear and spread easily and quickly across the region. Also in Thailand, asymptomatic gonorrhea prevalence was found to be higher among HIV-infected individuals who are less likely to have the infection diagnosed and treated effectively^23,24^. This may compromise early diagnosis and treatment of multi drug resistance *N. gonorrhoeae* infection.

In 2016, the Antimicrobial Resistance and Stewardship Research Unit, at the Faculty of Medicine, Chulalongkorn University in collaboration with the Thai Red Cross AIDS Research Centre began investigating a possible *N. gonorrhoeae* infection with resistance or reduce susceptibility to ceftriaxone in Thailand. Here, we first report two nearly identical isolates from heterosexual men with gonococcal infections, each sharing the similar mutation in the *penA* alleles, that demonstrate decreased susceptibility to ceftriaxone and remain susceptible to azithromycin.

## Results

### *N. gonorrhoeae* clinical isolates from urethral swabs in male patients had reduced susceptibility to ceftriaxone

Among the 134 *N. gonorrhoeae* clinical isolates collected during 2016–2019, two isolates (NG-083 and NG-091) which were isolated in 2017 from urethral swabs in male heterosexual patients at Thai Red Cross AIDS Research Centre, Anonymous Clinic, had reduced susceptibility to ceftriaxone (agar dilution MICs of 0.125 mg/L). Both isolates were resistant to penicillin G, tetracycline, ciprofloxacin, and gentamicin, while remaining susceptible to azithromycin (Table 1). The patients were a 30 and 32-year-old long term foreign resident men (South African and Australian) in Thailand, symptomatic with dysuria, or penile discharge at the time of specimen collection, and negative for HIV. Both were isolated cases and no relation to each other epidemiologically. One patient develops symptoms one week after unprotected oral sex with a female, while the second patients origin of infection was not disclosed. Both were treated for gonorrhea with Ceftriaxone 250 mg, and Azithromycin 250 mg as per current CDC guidelines^5^. Patients di not return to the clinical follow-up and therefore unable to be verified as having been cured or not.

**Table 1:** Patients summery and antimicrobial susceptibility profile against the *N. gonorrhoeae* isolates with reduced susceptibility to ceftriaxone (n = 2), Thailand, 2016–2018.

Patient Number	Isolate Number	Demographics	Sexual Preference	Healthcare Setting	Symptoms	Treatment	HIV Status	MIC (mg/L)	Biofilm Category	MBEC (mg/L)
83	NG-083	30-years-old South African Male	Heterosexual	Anonymous Clinic	Dysuria and Penile Discharge	Ceftriaxone 250 mg IM, + Azithromycin 250 mg orally twice daily × 14 d	Negative	Penicillin G 2 Tetracycline 4 Ciprofloxacin 4 Azithromycin 1 Cefixime 0.125 Ceftriaxone 0.125 Gentamicin 16 Fosfomycin 16 Ertapenem 1	Strong	Ceftriaxone 128 Azithromycin 256
91	NG-091	32-years-old Australian Male	Heterosexual	Anonymous Clinic	Penile Discharge	Ceftriaxone 250 mg IM, + Azithromycin 250 mg orally twice daily × 14 d	Negative	Penicillin G 2 Tetracycline 4 Ciprofloxacin 4 Azithromycin 0.5 Cefixime 0.125 Ceftriaxone 0.125 Gentamicin. 32 Fosfomycin 16 Ertapenem. 1	Strong	Ceftriaxone 128 Azithromycin 256

*HIV* human immunodeficiency viruses, *MIC* minimum inhibitory concentration, *IM* intramuscular, *MBEC* minimal biofilm eradication concentrations were categorized as responsive reaching about 90% of the total non-viable cells within a given antibiotic concentration range.

### *N. gonorrhoeae* isolates with reduced susceptibility to ceftriaxone are associated with alterations in PBP2, PBP1, PorB, MtrR, and mtrR promoter region

**Figure 1:**
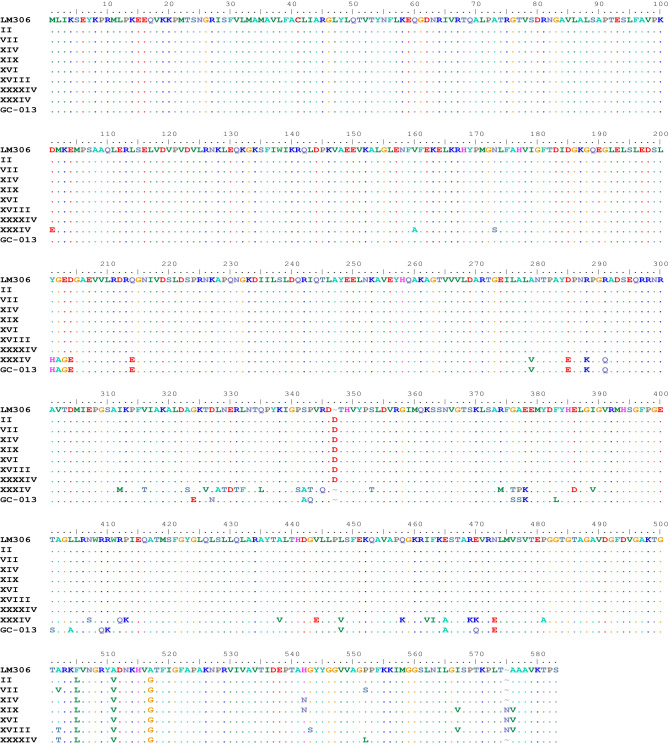
The amino acid sequence alignments of different PBP2 patterns of 9 N*. gonorrhoeae* isolates including nonmosaic PBP2 pattern II, VII, XIV, XIX, XVI, XVIII, XXXXIV, mosaic XXXIV, and new mosaic pattern (GC-013) were compared with the wild type PBP2 of *N. gonorrhoeae* strain LM306 (GenBank accession no. AAA25463).

**Figure 2:**
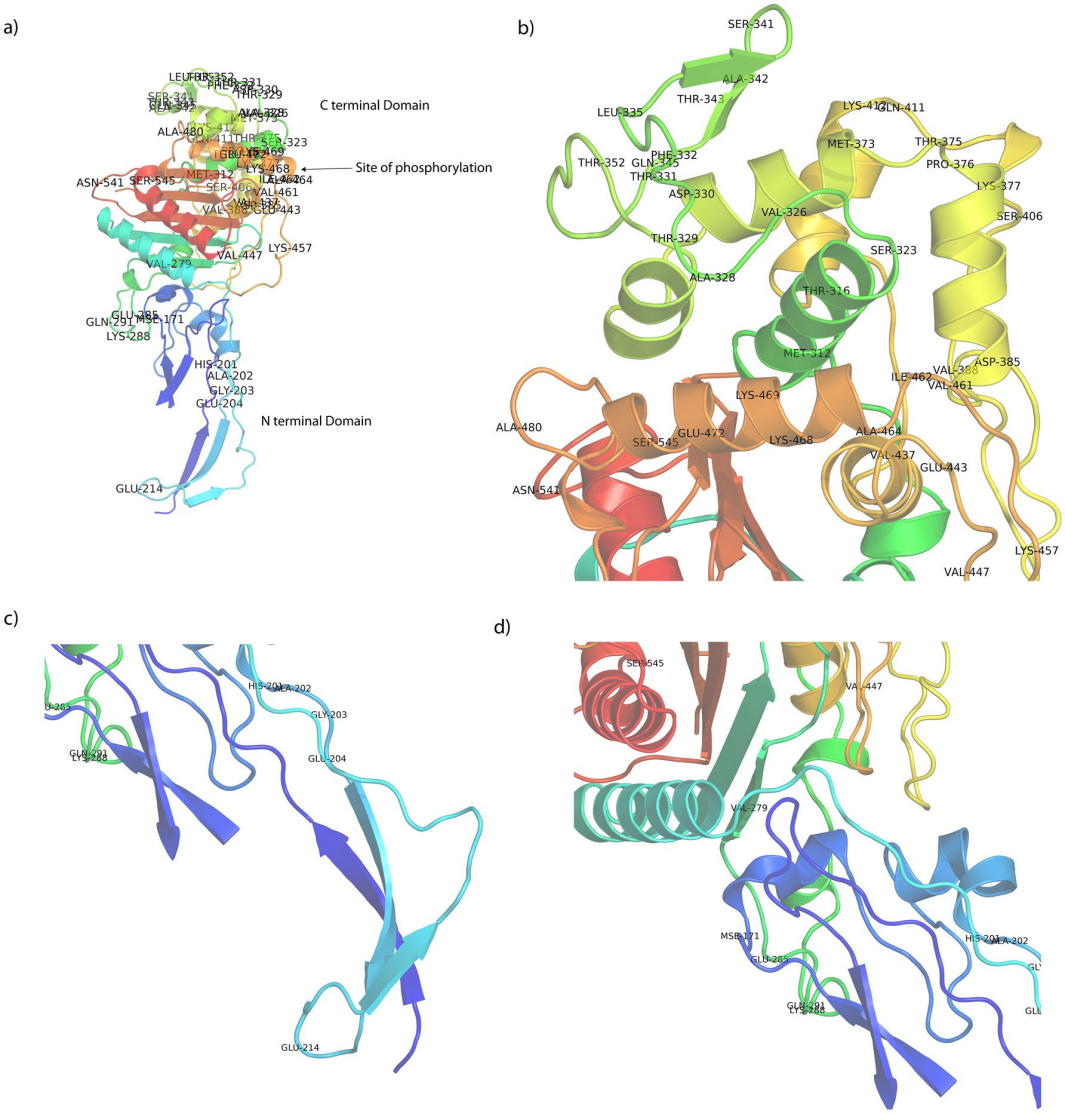
The structure of mosaic XXXIV PBP2 variant of *N. gonorrhoeae* clinical isolate (**a**) showing the location of important mutations and the active site sequence motifs, enlarge of (**b**) C terminal domain (**c**) N terminal domain (**d**) Site of phosphorylation. Mutations were modeled by PyMOL [DeLano, 2002] (The PyMOL Molecular Graphics System, Version 1.2r3pre, Schrödinger, LLC.).

The isolates NG-083 and NG-091 with reduced susceptibility to ceftriaxone harbor specific ceftriaxone resistance patterns (Table 2). NG-083 and NG-091 had an L421P substitution in PBP1, the mosaic PBP2 patterns XXXIV (Fig. 1) (Supplementary Figs. S2 and S3), an adenine deletion in the 13-bp inverted repeat sequence of the *mtrR* promoter region, an H105Y substitution in the MtrR repressor, and G120K and A121N substitutions in PorB porin. Mosaic XXXIV PBP2 variants contain up to 52 amino acid alterations compared with the wild-type PBP2 (Table 3). Figure 2 summarises the mosaic XXXIV PBP2 variant showing the location of all amino acid alterations drawn in Pymol using the crystal structure of a soluble form of *N. gonorrhoeae* wild-type PBP2 (extracted from rcsb.org/PDB: 3EQU).

**Table 2:** Mutations in resistance determinants and NG-MAST sequence types of the *N. gonorrhoeae* clinical isolates with reduced susceptibility to ceftriaxone (n = 2), Thailand, 2016–2018.

Patient number	Isolate number	Sexual Preference	Culture Site	Resistance Determinants					NG-MAST		
				PBP2	PBP1	*mtrR* promoter	MtrR	PorB	*por*	*tbpB*	ST
83	NG-083*	Male Heterosexual	Urethra	XXXIV	L421P	A del	H105Y	G120K, A121N	908	1180	ST7235
91	NG-091*	Male Heterosexual	Urethra	XXXIV	L421P	A del	H105Y	G120K, A121N	1914	New allele	New ST

*MIC* minimum inhibitory concentration, *WT* wild type, *New* new mosaic pattern, *PBP2* penicillin binding protein 2, *PBP1* penicillin binding protein 1, *mtrR*, *mtrR* promoter, *MtrR* MtrR repressor, *PorB* PorB porin, *NG-MAST* *N. gonorrhoeae* multi-antigen sequence typing, *NG-STAR* *Neisseria gonorrhoeae* Sequence Typing for Antimicrobial Resistance ST, sequence type, *A del* adenine deletion.

*N. gonorrhoeae isolates with reduced susceptibility to ceftriaxone.

**Table 3:** The amino acid residues in wild type *N. gonorrhoeae* Penicillin- binding proteins (PBPs) and mutative in Penicillin- binding proteins (mPBPs) in *N. gonorrhoeae* clinical isolates NG-083 and NG-091 with reduced susceptibility to ceftriaxone (n = 2), Thailand, 2016–2018.

Residue number	Original PDB:3EQU	Mutative PDB: 3EQU	Residue number	Original PDB:3EQU	Mutative PDB: 3EQU
101	ASP	GLU	374	AGR	MET
160	VLA	ALA	376	GLY	THR
173	ASN	SER	377	ALA	PRO
201	TYR	HIS	378	GLU	LYS
202	GLY	ALA	386	GLU	ASP
203	GLU	GLY	389	ILE	VAL
204	ASP	GLU	406	ASN	SER
214	GLN	GLU	412	ARG	GLN
279	ALA	VAL	413	PRO	LYS
285	ASP	GLU	438	ALA	VAL
288	ARG	LYS	444	VAL	GLU
289	ARG	GLN	448	LEU	VAL
312	ILE	MET	458	GLN	LYS
316	VAL	THR	462	ILE	VAL
323	ALA	SER	463	PHE	ILE
326	THR	VAL	465	GLU	ALA
328	LEU	ALA	469	ARG	LYS
329	ASN	THR	470	GLU	LYS
330	GLU	ASP	473	ASN	GLU
331	ARG	THR	481	PRO	ALA
332	LEU	PHE	505	PHE	LEU
335	GLN	LEU	511	ALA	VAL
341	PRO	SER	513	ASN	TRY
342	SER	ALA	541	HIS	ASN
343	PRO	THR	545	GLY	SER
345	ARG	GLN			
353	SER	THR

### *N. gonorrhoeae* isolates with reduced susceptibility to ceftriaxone belonged to different sequence types (STs)

Isolate NG-083 belonged to ST7235 (Table 2) and NG-091 had a new allele in *tbpB* and new ST according to the NG-MAST. However, both NG-083 and NG-091 have same ST-90 sequence types according to the NG-STAR (Table 4).

**Table 4:** Mutations in resistance determinants and NG-STAR sequence types of *N. gonorrhoeae* clinical isolates with reduced susceptibility to ceftriaxone (n = 2), Thailand, 2016–2018.

Patient number	Isolate number	Sexual preference	Culture site	Locus	Allele type	Amino acid alteration	NG-STAR Type
83,91	NG-083, NG-091	Male Heterosexual	Urethra	*penA*	34.001	*penA* Type XXXIV Mosaic with N513Y	90
				*mtrR*	1	-35A Del with H105Y	
				*porB*	11	G120K, A121N	
				*ponA*	1	L421P	
				*gyrA*	1	S91F, D95G	
				*parC*	3	S87R	
				23S rRNA	100	Wild Type	

*MIC* minimum inhibitory concentration, *WT* wild type, *New* new mosaic pattern, *PBP2* penicillin binding protein 2, *PBP1* penicillin binding protein 1, *mtrR* *mtrR* promoter, *MtrR* MtrR repressor, *PorB* PorB porin, *NG-STAR* *Neisseria gonorrhoeae* sequence typing for antimicrobial resistance ST, sequence type, *A Del* adenine deletion.

*N. gonorrhoeae isolates with reduced susceptibility to ceftriaxone.

### Ceftriaxone weakly occupies the active site of mosaic XXXIV penicillin-binding protein 2 (PBP2) variant in *N. gonorrhoeae* isolates with reduced susceptibility to ceftriaxone

**Figure 3:**
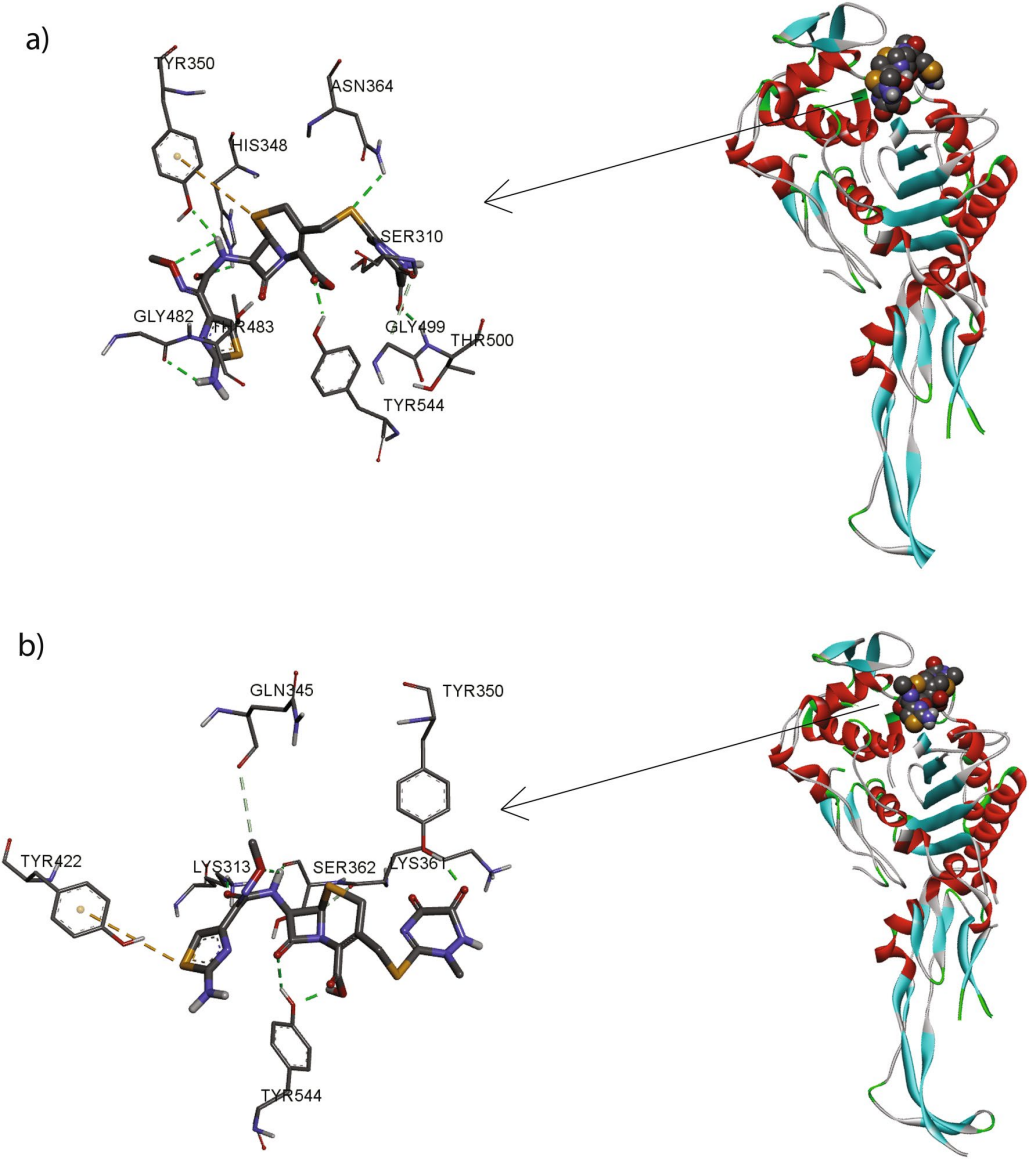
Molecular docking of ceftriaxone in the active site of a) wild type PBP2 and b) mosaic XXXIV PBP2 variant. The standard macromolecule was extracted from rcsb.org/ (PDB: 3EQU) and the mutations were modeled by PyMOL [DeLano, 2002] (The PyMOL Molecular Graphics System, Version 1.2r3pre, Schrödinger, LLC.).

**Figure 4:**
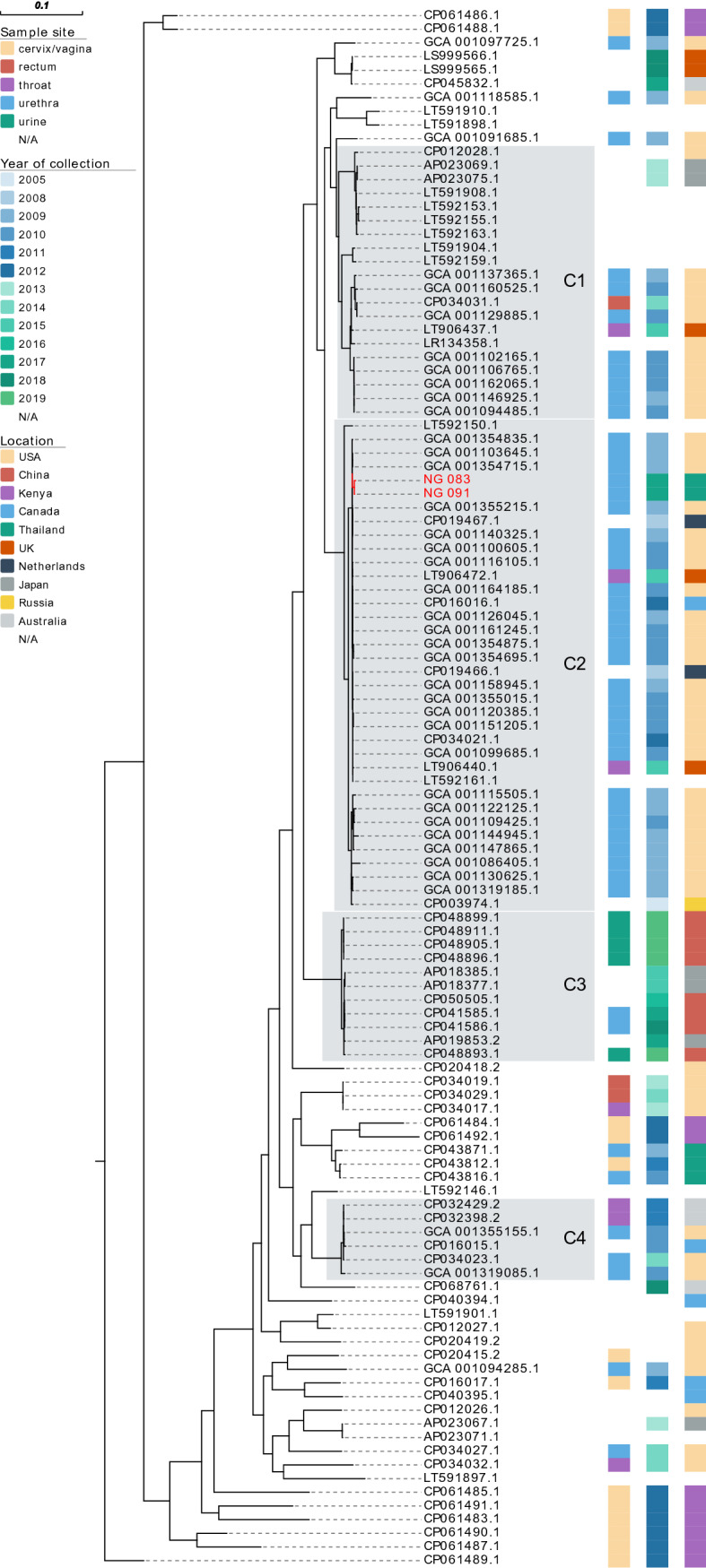
Phylogenetic analyses based on core genome from draft genomes of *Neisseria gonorrhoeae* NG-083 and NG-091 clinical isolated in Thailand and from varying *N*. gonorrhoeae WGS investigations conducted elsewhere (available on NCBI database) were determined for the number of single nucleotide polymorphisms (SNPs) by Core-Genome SNP Analysis. C1-C4 represent major clusters.

Ceftriaxone (binding energy: − 6.64 kcl/mol, Ki: 13.6 μM) has formed three hydrogen bonds with three different amino acids (Fig. 3). The NH of Thr500 has formed an H-bond with 1,2,4-triazine ring of ceftriaxone at a distance of 1.828 Å, within the *β*3–*β*4 loop of standard PBP2 binding site. The OH of Tyr350 has formed a hydrogen bond with NH group near the cephalosporin ring (O–HN) at distance of 2.219 Å. The C=O group of carboxylic acid has formed an acyl-enzyme complex by making an H-bond with OH of Tyr 544 with a distance of 2.019 Å and completely occupied the catalytic triad located at the N-terminal end of helix α11. In case of mosaic XXXIV PBP2 variant, the binding energy of ceftriaxone was found to be -5.92 kcl/mol with 45.63 μM as Ki value, which is far less than the standard PBP2. The ceftriaxone has formed two hydrogen bonds and one π–π interaction with the altered protein (Fig. 4). The OH phenol group of tyr350 has formed an h-bond with ceftriaxone (OH—- -O) at a distance of 1.894 Å. The C=O group near the cephalosporin ring has formed a H-bond with the NH group of Lys313 at a distance of 1.894 Å, which confirms the binding in the active site. The π-µ interaction was observed between 1,2,4-triazine ring of ceftriaxone and the phenolic ring of Tyr422 at a distance of 14.323 Å, which helps to understand the rotation of the molecule within the active site.

### *N. gonorrhoeae* isolates with reduced susceptibility to ceftriaxone are closely related with urethra isolates from male patients from Las Vegas

A rooted-ML phylogenetic tree demonstrated four major cluster (C1–C4) (Fig. 4 and Supplementary Fig. S4), each of which contained at least six isolates, using 3% substitutions per site as a cluster genetic distance (8). Cluster C2, the largest cluster, involved 36 *N. gonorrhoeae* isolates from the USA, Netherlands, the UK, and Canada, and included two ceftriaxone-reduced susceptible isolates found in our study (NG-083 and NG-091) from two foreign male patients in Thailand in 2017. The isolates are closely related with two *N. gonorrhoeae* isolated from the urethra of male patients from Las Vegas in the USA in 2009 under BioProject accession no PRJEB2999, which differed by 2 core SNPs (accession no GCA_001103645.1 and GCA_001354715.1).

### Ceftriaxone plus azithromycin combination display no synergistic but bactericidal activity against *N. gonorrhoeae* isolates with reduced susceptibility to ceftriaxone

**Figure 5:**
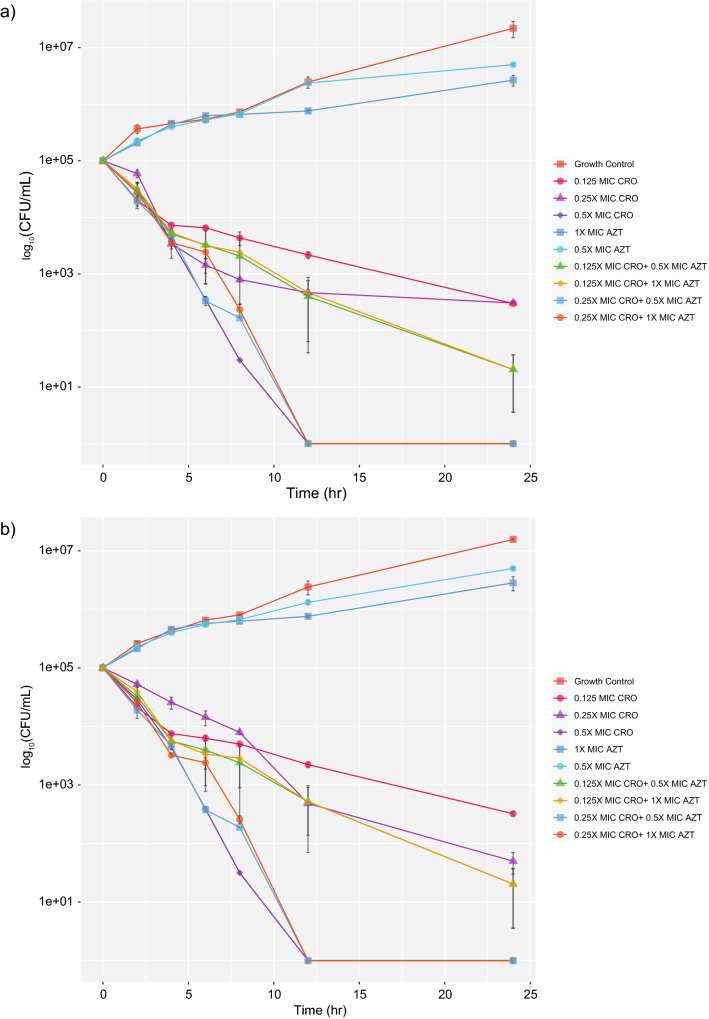
Time-kill curve of ceftriaxone plus azithromycin against *N. gonorrhoeae* isolates with reduced susceptibility to ceftriaxone (**a**) NG-083 and (**b**) NG-091.

Synergistic and antagonistic effects were not found in all antibiotic combinations against both isolates (Table 5). Additive effects were found with ceftriaxone plus azithromycin, fosfomycin, or gentamicin. Indifferent was found in ceftriaxone plus ertapenem. At 0.5X MIC of ceftriaxone, bacterial growth was rapidly inhibited within 8 to 12 h in both isolates, but the concentration at 0.125X and 0.25X MIC was unable to inhibit bacterial growth within 24 h (Fig. 5). However, 0.125X MIC of ceftriaxone plus 0.5X or 1X MIC of azithromycin, and 0.25X MIC of ceftriaxone plus 0.5X or 1X MIC of azithromycin showed effective killing against NG-083 and NG-091.

**Table 5:** Summary of antibiotic combinations against *N. gonorrhoeae* clinical isolates NG-083 and NG-091 with reduced susceptibility to ceftriaxone (n = 2), Thailand, 2016–2018.

Isolate no	Resistance Determinants					NG-MAST	FICI index			
	PBP2 pattern	PBP1	*mtrR* promoter	MtrR	PorB porin		CRO:AZT	CRO:FOS	CRO:GEN	CRO:ETP
NG-083	XXXIV	L421P	A del	H105Y	G120K, A121N	ST7235	0.75 (A)	1.00 (A)	1.00 (A)	1.50 (I)
NG-091	XXXIV	L421P	A del	H105Y	G120K, A121N	New ST	0.74 (A)	1.00 (A)	1.00 (A)	1.25 (I)

*AZT* azithromycin, *CRO* ceftriaxone, *ETP* ertapenem, *FOS* fosfomycin, *GEN* Gentamicin, *A* additive, *I* indifferent, *FICI* Fractional Inhibitory Concentration Index.

### *N. gonorrhoeae* isolates with reduced susceptibility to ceftriaxone produce strong biofilms with ceftriaxone tolerance

**Figure 6:**
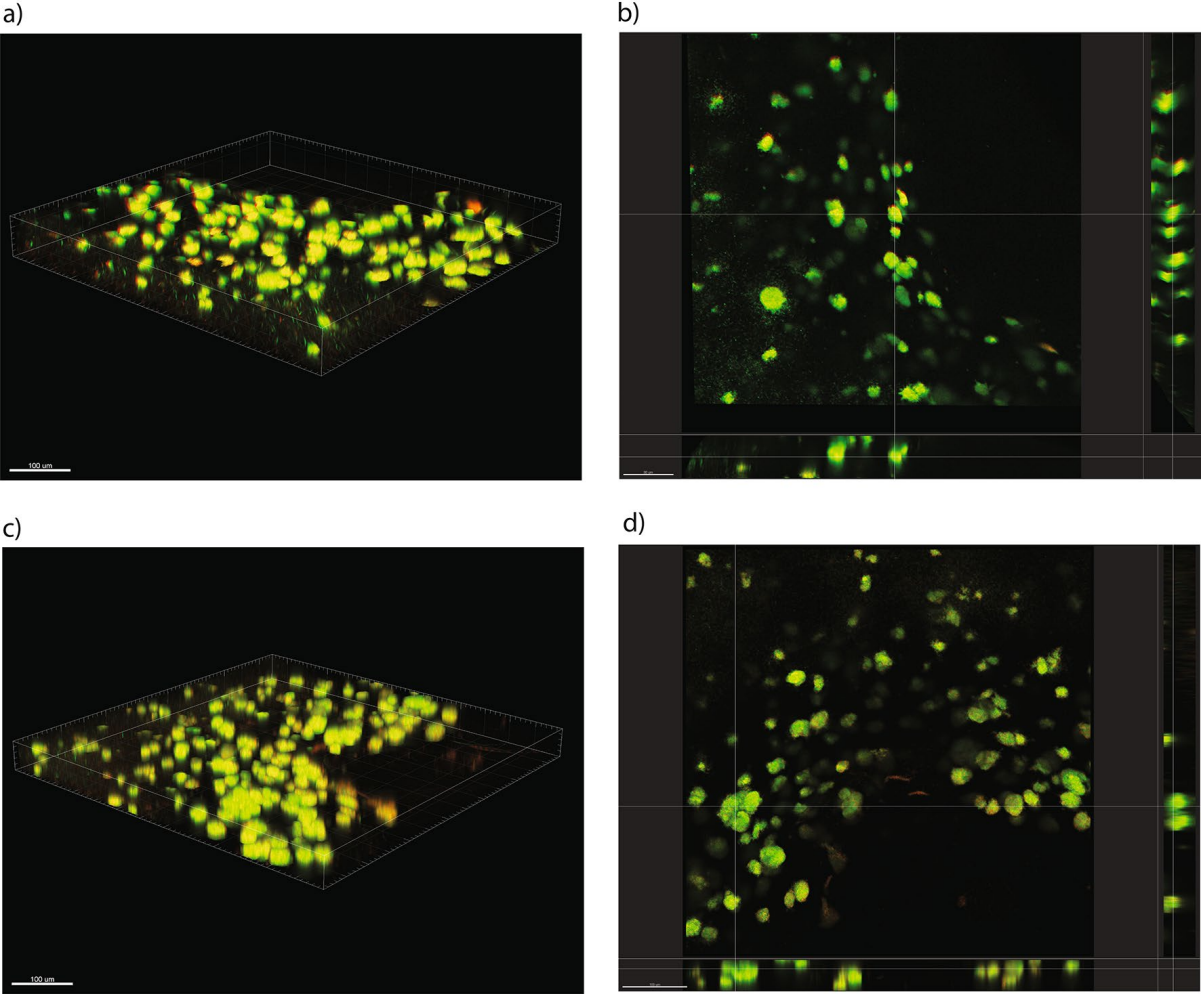
Confocal imaging analysis (3D and cross sectional) of biofilm structure of *N. gonorrhoeae* isolates (**a**,**b**) NG-083 and (**c**,**d**) NG-091treated with MIC concentration (0.125 mg/L) of ceftriaxone.

**Figure 7:**
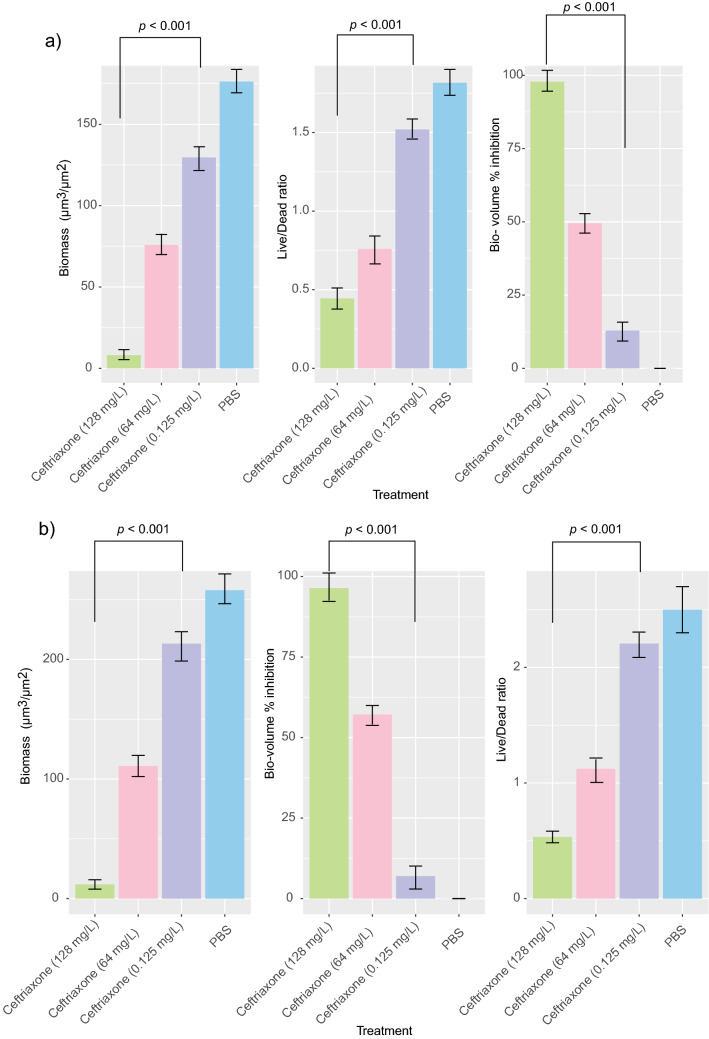
Effects of different concentration of ceftriaxone on *N. gonorrhoeae* isolates (n = 2). Isolate NG-083; (**a**) in vitro biomass (**b**) in vitro Live/Dead cell ratio (**c**) in vitro bio-volume inhibition and isolate NG-091; (**d**) in vitro biomass (**e**) in vitro bio-volume inhibition (**f**) in vitro Live/Dead cell ratio.

The isolates NG-083 biofilm had significantly more biomass than the NG-091 biofilm (P = 0.004) (Fig. 6). Both isolates were strong biofilm producers with an atypical ellipse-shaped structure or clumps, and well tolerated to a ceftriaxone concentration up to 128 mg/L (Fig. 7). The analysis showed that the live/dead bacterial ratio of NG-083 and NG-091 biofilms treated with ceftriaxone MIC (0.125 mg/L) concentration was not different in comparison with non-treated group (P > 0.05) (Fig. 7). Both isolates displayed minimal biofilm eradication concentration for ceftriaxone around 128 mg/L concentration (Table 1).

## Discussion

We identified a pair of gonococcal isolates with decreased susceptibility to ceftriaxone, ciprofloxacin, tetracycline, penicillin G, gentamicin, and ertapenem, while remaining susceptible to azithromycin. Both isolates appeared to be unrelated, with for example two different sequence types according to the NG-MAST but same sequence type according to Ng-STAR. The genome sequencing analysis revealed alterations in PBP2, PBP1, PorB, MtrR, and *mtrR* promoter region in both isolates. Molecular docking studies suggested ceftriaxone weakly occupies the active site of mosaic XXXIV PBP2 variant, which may explain the decreased ceftriaxone activity. Interestingly, both isolates with reduced susceptibility to ceftriaxone demonstrated strong biofilm production that was associated with ceftriaxone tolerance at concentrations higher than the MIC. Molecular epidemiology results revealed that both isolates display similarities with isolates from the UK, USA, and the Netherlands. The most effective combination was ceftriaxone plus azithromycin which showed bactericidal activity for both isolates.

This Thai isolates is concerning because two isolates from heterosexual male patients (NG-083 and NG-091) demonstrated decreased susceptibility to ceftriaxone with mosaic PBP2 pattern XXXIV, similar to isolates previously reported from USA^25^, Austria^26^, Japan^27^, France^17^, Spain^28^, and Canada^29^. Moreover, these two isolates are genetically closely related to two *N. gonorrhoeae* isolated from the urethra of male patients from Las Vegas in USA in 2009, which also exhibited reduced susceptibility to cefixime, with most of the isolates having mosaic *penA* allele XXXIV^30^. As given the 8 years between specimen collection of the Las Vegas isolates and two isolates in our study, it’s difficult to relate an epidemiological connection between these two and the Las Vegas isolates. However, Thailand is a popular getaway for sex tourism^3^, there is the possibility that these two isolates (NG-083 and NG-091) may have transmitted through travelers. However, because we did not have samples from other sexual partners of these two men to provide broader context, is difficult to established clear epidemiological relations.

To our knowledge, no isolates have previously been reported in Thailand with decreased ceftriaxone susceptibility. The data from the National Antimicrobial Resistance Surveillance Center, Thailand (NARST)^31^, and other studies from Thailand^32^ showed that 100% of *N. gonorrhoeae* isolates were susceptible to cefixime and ceftriaxone. A report from the United Kingdom documented a gonorrhea treatment failure with the isolate exhibiting a ceftriaxone MIC = 0.25 µg/mL and azithromycin MIC = 1 µg/mL^12,33^. In comparison, our isolates demonstrate similar susceptibility to azithromycin, while their ceftriaxone MICs were only a single dilution lower than the ceftriaxone MIC from the UK isolate, suggesting that if the strains identified in Thailand develop higher ceftriaxone MICs, they may be capable of causing treatment failure. It is uncertain whether current gonococcal treatment of ceftriaxone plus azithromycin may have contributed to the development of higher ceftriaxone MICs and widespread transmission of such strains^34^. In addition, the improper use or misuse of antibiotics, and over-the-counter drug usage in Thailand, may have contributed to the development of higher ceftriaxone MICs^35^. It is possible that the persistence of such isolates within biofilm, seen with infections involving higher MICs, reflected a slower killing of *N. gonorrhoeae* and may severely complicate gonorrhea treatment.

The threat of multidrug-resistant gonorrhea leading to treatment failure in Thailand with the current recommended dual therapy remains present. Our findings emphasized the value of the CDC recommendations for laboratories to maintain culture-based methods to detect antimicrobial-resistant *N. gonorrhoeae*, particularly for patients with possible treatment failure. In addition, we also emphasized the potential value of genomic monitoring to detect antimicrobial-resistant *N. gonorrhoeae*. It is important that clinicians be on high alert so that treatment failures can be identified and reported promptly to the local department of health. Rapid detection and effective treatment may prevent sequelae, allow partners to be identified and treated in a timely manner, and prevent or slow further transmission of resistant strains. Furthermore, given the study findings, we advised continued practice of dual therapy with ceftriaxone 250 mg plus azithromycin 1gm in Thailand. However, it is necessary to have new strategies such as resistance guided therapy for *N. gonorrhoeae* infections and contact tracing to identify the origin of the gonococcal infections as preventive measures. Finally, there are urgent need for development of new or combination therapies to tackle the multidrug resistant *N. gonorrhoeae* infections.

The strengths of this study are that all infections were diagnosed by culture, which has 100% specificity for gonorrhea.

## Methods

### *N. gonorrhoeae* clinical isolates

134 isolates collected from urethral swabs of patients with positive *N. gonorrhoeae* infections at Thai Red Cross Anonymous Clinic, Thai Red Cross AIDS Research Centre, and King Chulalongkorn Memorial Hospital, Bangkok, Thailand, from patients with gonococcal infections during 2016–2019. For culture preservation, all isolates were grown on TM medium (GC agar base supplemented with 1% haemoglobin, 1% IsoVitaleX, and vancomycin, colistin sulfate, and nystatin selective supplement (Oxoid, United Kingdom). All *N. gonorrhoeae* isolates were preserved at − 80 °C. *N. gonorrhoeae* ATCC 49226 reference strain was used for quality control of all phenotypic and molecular characterizations.

### Phenotypic antimicrobial susceptibility testing

The isolates obtained from patients were analysed using both broth micro-dilution and agar plate dilution methods (Supplementary methods). MIC interpretive criteria were in accordance with the Clinical and Laboratory Standards Institute (CLSI)^36^or European Committee on Antimicrobial Susceptibility Testing (EUCAST)^37^ when available. In the present study in vitro decreased susceptibility to ceftriaxone was defined as having an MIC of > 0.064–0.125 mg/L.

### Molecular epidemiological typing

The presence of *carA* and *orf1* genes were detected by specific PCR primers (Supplementary methods Table S1) (BioDesign, Thailand) to confirm *N. gonorrhoeae* identification as described previously^38,39^.

### Detection and characterization of ceftriaxone resistance mechanisms

The ceftriaxone resistance mechanisms in *N. gonorrhoeae* isolates were investigated by *penA*, *mtrR*, *ponA*, and *porB* genes using PCR and automated DNA sequencing (1st BASE Inc, Malaysia) (Supplementary methods Table S3). The PCR products of genes were purified using HiYieldTMGel/PCR fragments extraction kit as described by the manufacturer (RBC Bioscience, Taiwan). Sequencing was conducted using BigDye terminator cycling conditions.

### Clonal studies of *N. gonorrhoeae*

The clonality of *N. gonorrhoeae* was initially determined by NG-MAST (http://www.ng-mast.net/) as previously described^40^. The internal fragments of 2 highly polymorphic antigen-encoding loci including outer membrane proteins (*por*) and transferrin binding protein unit B (*tbpB*)^40^ were amplified, purified (HiYieldTMGel/PCR) and sequenced (Supplementary methods Table S4) as previously described^40^. However, because NG-MAST no longer assigns new STs, the two *N. gonorrhoeae* with reduced susceptibility to ceftriaxone were further analyzed with NG-STAR. Sequence of 7 genes (*penA*, *mtrR*, *porB*, *ponA*, *gyrA*, *parC* and 23S rRNA) were extract from genome sequence of NG-083 and NG-091 isolates, then the sequence were analyzed using NG-STAR database (https://ngstar.canada.ca/alleles/query?lang=en). Preparation of sequencing reactions were performed by the 1st BASE Inc, Malaysia, as described above.

### Whole-genome sequencing and genome assembly

The genomes of two *N. gonorrhoeae* with reduced susceptibility to ceftriaxone were sequenced using Illumina sequencing platform (2 × 150 paired-end, Illumina, Inc., USA). The genomic DNA was extracted with the PureLink PCR Purification kit and quantified in a Qubit Fluorometer (Invitrogen), following the manufacturer's recommendations. Libraries were sequenced on a NovaSeq 6000. The low-quality and adapter bases were trimmed using Skewer v0.2.2 (https://github.com/relipmoc/skewer). The quality of pre- and postprocessed reads was assessed with the FastQC tool, v0.11.8 (http://www.bioinformatics.babraham.ac.uk/projects/fastqc/). The resulting high-quality reads were assembled de novo using Unicycler v0.4.8 (PMID: 28594827). The quality of genome sequences was checked using QUAST v5.0.2 (PMID: 23422339) and annotated by DFAST v1.2.6 (PMID: 29106469).

### Phylogenetic analyses

The core genome from draft genomes of *N. gonorrhoeae* NG_83 and NG_91 clinical isolated in Thailand and from other *N. gonorrhoeae* WGS investigations conducted elsewhere (available on NCBI database) were determined for the number of single nucleotide polymorphisms (SNPs) by Core-Genome SNP Analysis. A reference was randomly selected among the genome sequences to generate a core genome alignment and a phylogenetic tree was constructed using a core SNP alignment. Draft genomes of *N. gonorrhoeae* were aligned following the detection and filtration of recombinant regions using Parsnp v1.2^41^ and Gubbins v2.4.1^42^. Maximum-likelihood (ML) trees were generated by RAxML v8.2.12^43^ using ASC_GTRGAMMA model of rate heterogeneity with the Lewis correction for ascertainment bias^44,45^. Best-scoring ML tree was visualized and annotated as a phylogenetic tree using FigTree v1.4.4 and Evolview v2^46–48^ (Supplementary File GC_114RV2parsnp).

### Sequence analysis

The nucleotide sequences and deduced amino acid sequences were analyzed with the online software available at the National Center for Biotechnology Information (NCBI; https://www.ncbi.nlm.nih.gov/BLAST), and ExPASy (www.expasy.org). Multiple sequence alignment was analyzed by Multalin (http://multalin.toulouse.inra.fr/multalin). The nucleotide and deduced amino acid sequences identified in this study including *penA*, *ponA*, *mtrR*, and *porB* genes were compared with the corresponding sequences in the genome sequenced of *N. gonorrhoeae* reference strain FA1090 (GenBank accession number AE004969). The nucleotide sequences of 2 highly polymorphic antigen-encoding loci were analyzed and uploaded onto the NG-MAST database (http://www.ng-mast.net) to obtain the allele number and the sequence type (ST). Furthermore, two *N. gonorrhoeae* with reduced susceptibility to ceftriaxone were analyzed with NG-STAR (https://ngstar.canada.ca).

### Molecular docking

Molecular docking studies were carried out to understand binding mode analysis and orientation of ceftriaxone in the active site of PBP2 and compared with mosaic XXXIV PBP2 variant. The standard macromolecule was extracted from rcsb.org/ (PDB: 3EQU) and the mutations were modeled by PyMOL [DeLano, 2002] (The PyMOL Molecular Graphics System, Version 1.2r3pre, Schrödinger, LLC.) (Supplementary methods).

### Antibiotic synergy analysis using microdilution checkerboard assay

The synergistic activities of antibiotics were screened against 2 strains of *N. gonorrhoeae* isolates with reduced susceptibility to ceftriaxone using checkerboard method (Supplementary methods).

### Time kill assay

Two strains of *N. gonorrhoeae* with reduced susceptibility to ceftriaxone that showed the best synergistic activity by checkerboard method was confirmed using time-kill assay. All experiments were performed at least three times (Supplementary methods).

### Biofilm formation, quantification and classification

Biofilm formation in a 96-well-microtitre-plate format was performed as described previously^49–51^ (Supplementary methods). Two methods^52,53^ were used to quantify and classify the biofilm by Crystal Violet staining and and confocal laser scanning microscopy, performed in triplicate, and repeated three times.

### Minimal biofilm eradication concentrations (MBEC)

Minimal biofilm eradication concentrations (MBEC) were established as described previously^49–51^ by adding the serially diluted antibiotics to mature biofilms and incubating at 37 °C for 24 h and then staining with PrestoBlue (Thermo Fisher Scientific). All experiments were performed in triplicate and repeated three times.

### Statistical analyses

Fisher's exact test (two-tailed) was used to verify the association between mutations in resistance determinants and penicillin resistance in *N. gonorrhoeae* isolates by using the R statistical package. Biofilm experiments were analyzed by confocal laser scanning microscope (biomass, Live/Dead ratio and biovolume inhibition). MATLAB-based tool PHLIP (without connected volume filtration) were used to calculate descriptive parameters of biofilms (including biovolume, substratum coverage, area-to-volume ratio, spatial spreading and 3D colocalization) from the integrated total of each individual slice of a thresholded z-stack as described previously^49,50,54^. The calculation of the different proportions of green (live bacteria) as well as red and yellow/colocalized (dead bacteria) biovolumes from the analyzed stacks were using the 'colocalization in 3D' value and the parameters 'red', 'green', and 'total biovolume' (in μm^3^) generated by the PHLIP software as described previously^48,49,53^. A biofilm was considered affected by an antibiotic within the given concentration range when there is a constant increase in the red + yellow (RY) biovolume fraction within the given antibiotic concentration range and this fraction is at least 80% of the total biovolume. The data were compared by either unpaired two-tailed Student’s t-test or unpaired two-tailed Mann–Whitney’s U test. Statistical significance was accepted at *p* < 0.05, *p* < 0.01, *p* < 0.001, and *p* < 0.0001.

### Nucleotide sequence accession numbers

Genome assemblies of NG-083 and NG-091 isolates were submitted to the NCBI Database under BioSamples of SAMN20286673 and SAMN20286674. These two BioSamples were submitted under BioProject PRJNA747638 (https://www.ncbi.nlm.nih.gov/bioproject/747638), which the author sequence were the same with the author manuscript.

### Ethics approval

The study protocol was approved (IRB No. 396/60, COA No. 715/2017) by the Institutional Review Board of the Faculty of Medicine, Chulalongkorn University, Bangkok, Thailand, and was performed in accordance with the ethical standards as laid down in the 1964 Declaration of Helsinki and its later amendments and comparable ethical standards.

### Informed consent

For this retrospective study of anonymous clinical isolates, the requirement for informed consent from patients was waived by the Institutional Review Board of the Faculty of Medicine, Chulalongkorn University, Bangkok, Thailand (IRB No. 396/60, COA No. 715/2017).

## Supplementary Information

Supplementary Information 1.

Supplementary Information 2.

Supplementary Information 3.

Supplementary Information 4.

Supplementary Information 5.

Supplementary Information 6.

## Data Availability

Data generated and analysed during this study are included in this published article and its Supplemental information file. Additional clinical details available upon reasonable request from corresponding author TC.
