# Supplementary material for: Multidrug-resistant Neisseria gonorrhoeae infection in heterosexual men with reduced susceptibility to ceftriaxone, first report in Thailand
Source: Sci Rep. 2021 Nov 4;11:21659. doi: 10.1038/s41598-021-00675-y (PMC8569152; doi:10.1038/s41598-021-00675-y)

Figure 1. Relationship between antibiotic concentration, patients age and sexual preferences of *N. gonorrhoeae* clinical isolates Thailand, 2016-2018 a) PEN, penicillin G b) TET, tetracycline c) CIP, ciprofloxacin d) AZT, azithromycin e) CFM, cefixime f) CRO, ceftriaxone g) FOS, fosfomycin h) GEN, gentamicin i) ETP, ertapenem

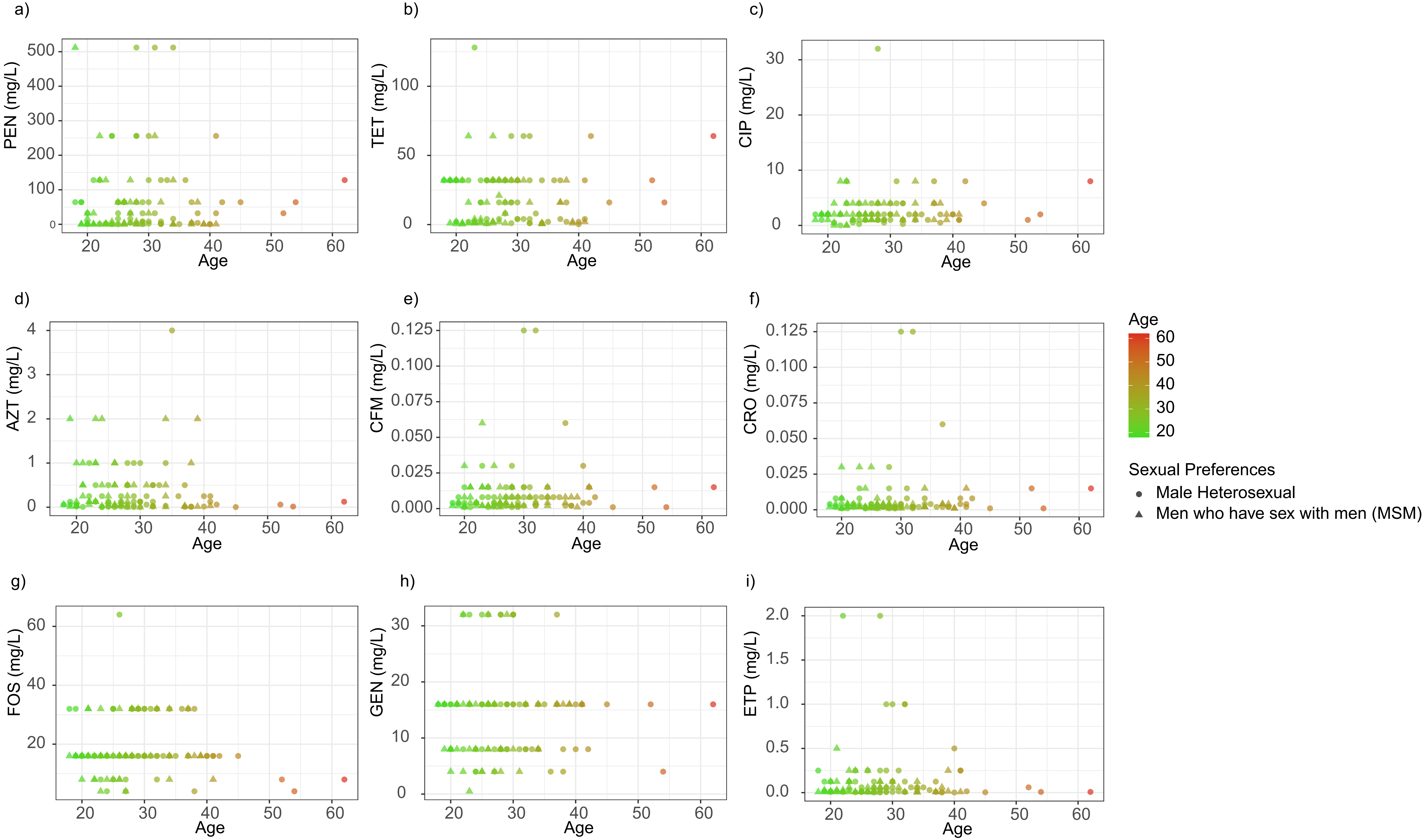

Supplement: Supplementary file 1 — Supplementary Information 1. [file 41598_2021_675_MOESM1_ESM.pdf]
