# Supplementary material for: Multidrug-resistant Neisseria gonorrhoeae infection in heterosexual men with reduced susceptibility to ceftriaxone, first report in Thailand
Source: Sci Rep. 2021 Nov 4;11:21659. doi: 10.1038/s41598-021-00675-y (PMC8569152; doi:10.1038/s41598-021-00675-y)
Supplement: Supplementary file 2 — Supplementary Information 2. [file 41598_2021_675_MOESM2_ESM.pdf]

Supplementary Figure 2 Alignment for amino acid sequences of PBP2 of *N. gonorrhoeae* strain CG-013 and *N. gonorrhoeae* strain LM306 (GenBank accession no. AAA25463)

```
                 10        20        30        40        50        60        70        80        90       100
        ....|....|....|....|....|....|....|....|....|....|....|....|....|....|....|....|....|....|....|....|
M38900  MLIKSEYKPRMLPKEEQVKKPMTSNGRISFVLMAIAVLFAGLIARGLYLQTVTYNFLKEQGDNRIVRTQTLPATRGTVSDRNGAVLALSAPTESLFAVPK
GC-013  ..................................M.....C............................A..............................

                110       120       130       140       150       160       170       180       190       200
        ....|....|....|....|....|....|....|....|....|....|....|....|....|....|....|....|....|....|....|....|
M38900  EMKEMPSAAQLERLSELVDVPVDVLRNKLEQKGKSFIWIKRQLDPKVAEEVKALGLENFVFEKELKRHYPMGNLFAHVIGFTDIDGKGQEGLELSLEDSL
GC-013  D...................................................................................................

                210       220       230       240       250       260       270       280       290       300
        ....|....|....|....|....|....|....|....|....|....|....|....|....|....|....|....|....|....|....|....|
M38900  HAGEGAEVVLRDREGNIVDSLDSPRNKAPQNGKDIILSLDQRIQTLAYEELNKAVEYHQAKAGTVVVLDARTGEILALVNTPAYEPNKPGQADSEQRRNR
GC-013  ....................................................................................................

                310       320       330       340       350       360       370       380       390       400
        ....|....|....|....|....|....|....|....|....|....|....|....|....|....|....|....|....|....|....|....|
M38900  AVTDMIEPGSAIKPFVIAKALDADKTNLNERLNTQPYKIGPAQVRDTHVYPSLDVRGIMQKSSNVGTSKLSARFSSKEMYDLYHELGIGVRMHSGFPGES
GC-013  .......................E............................................................................

                410       420       430       440       450       460       470       480       490       500
        ....|....|....|....|....|....|....|....|....|....|....|....|....|....|....|....|....|....|....|....|
M38900  AGALRNWQKWRPIEQATMSFGYGLQLSLLQLARAYTALTHDGVLLPVSFEKQAVAPQGKRIFKASTARQVRELMVSVTEPGGTGTAGAVDGFDVGAKTGT
GC-013  ....................................................................................................

                510       520       530       540       550       560       570       580
        ....|....|....|....|....|....|....|....|....|....|....|....|....|....|....|....|..
M38900  ARKLVNGRYVDYKHVATFIGFAPAKNPRVIVAVTIDEPTANGYYGGVVTGPVFKQVMGGSLNILGVSPTKPLTNVAAVKTPS
GC-013  ..................................................................................
```

Supplementary Figure 3 Alignment for amino acid sequences of PBP2 of *N. gonorrhoeae* strain CG-013 and *N. meningitidis* strain M38900 (GenBank accession no. WP_118824975.1)

```
                10        20        30        40        50        60        70        80        90       100
        ....|....|....|....|....|....|....|....|....|....|....|....|....|....|....|....|....|....|....|....|
LM306   MLIKSEYKPRMLPKEEQVKKPMTSNGRISFVLMAMAVLFACLIARGLYLQTVTYNFLKEQGDNRIVRTQALPATRGTVSDRNGAVLALSAPTESLFAVPK
GC-013  ....................................................................................................

               110       120       130       140       150       160       170       180       190       200
        ....|....|....|....|....|....|....|....|....|....|....|....|....|....|....|....|....|....|....|....|
LM306   DMKEMPSAAQLERLSELVDVPVDVLRNKLEQKGKSFIWIKRQLDPKVAEEVKALGLENFVFEKELKRHYPMGNLFAHVIGFTDIDGKGQEGLELSLEDSL
GC-013  ....................................................................................................

               210       220       230       240       250       260       270       280       290       300
        ....|....|....|....|....|....|....|....|....|....|....|....|....|....|....|....|....|....|....|....|
LM306   YGEDGAEVVLRDRQGNIVDSLDSPRNKAPQNGKDIILSLDQRIQTLAYEELNKAVEYHQAKAGTVVVLDARTGEILALANTPAYDPNRPGRADSEQRRNR
GC-013  HAGE.........E................................................................V.....E..K..Q.........

               310       320       330       340       350       360       370       380       390       400
        ....|....|....|....|....|....|....|....|....|....|....|....|....|....|....|....|....|....|....|....|
LM306   AVTDMIEPGSAIKPFVIAKALDAGKTDLNERLNTQPYKIGPSPVRDTHVYPSLDVRGIMQKSSNVGTSKLSARFGAEEMYDFYHELGIGVRMHSGFPGET
GC-013  .......................E..N..............AQ...............................SSK....L.................S

               410       420       430       440       450       460       470       480       490       500
        ....|....|....|....|....|....|....|....|....|....|....|....|....|....|....|....|....|....|....|....|
LM306   AGLLRNWRRWRPIEQATMSFGYGLQLSLLQLARAYTALTHDGVLLPLSFEKQAVAPQGKRIFKESTAREVRNLMVSVTEPGGTGTAGAVDGFDVGAKTGT
GC-013  ..A....QK.....................................V................A....Q..E............................

               510       520       530       540       550       560       570       580
        ....|....|....|....|....|....|....|....|....|....|....|....|....|....|....|....|..
LM306   ARKFVNGRYADNKHVATFIGFAPAKNPRVIVAVTIDEPTAHGYYGGVVAGPPFKKIMGGSLNILGISPTKPLT~AAAVKTPS
GC-013  ...L.....V.Y............................N.......T..V..QV.........V.......NV.......
```
