# Supplementary material for: Multidrug-resistant Neisseria gonorrhoeae infection in heterosexual men with reduced susceptibility to ceftriaxone, first report in Thailand
Source: Sci Rep. 2021 Nov 4;11:21659. doi: 10.1038/s41598-021-00675-y (PMC8569152; doi:10.1038/s41598-021-00675-y)

Supplementary Figure 4 Phylogenetic analyses based on core genome from draft genomes of *Neisseria gonorrhoeae* NG-083 and NG-091 (Bootstrap score tree) clinical isolated in Thailand and from varying *N. gonorrhoeae* WGS investigations conducted elsewhere (available on NCBI database) were determined for the number of single nucleotide polymorphisms (SNPs) by Core-Genome SNP Analysis. C1-C4 represent major clusters

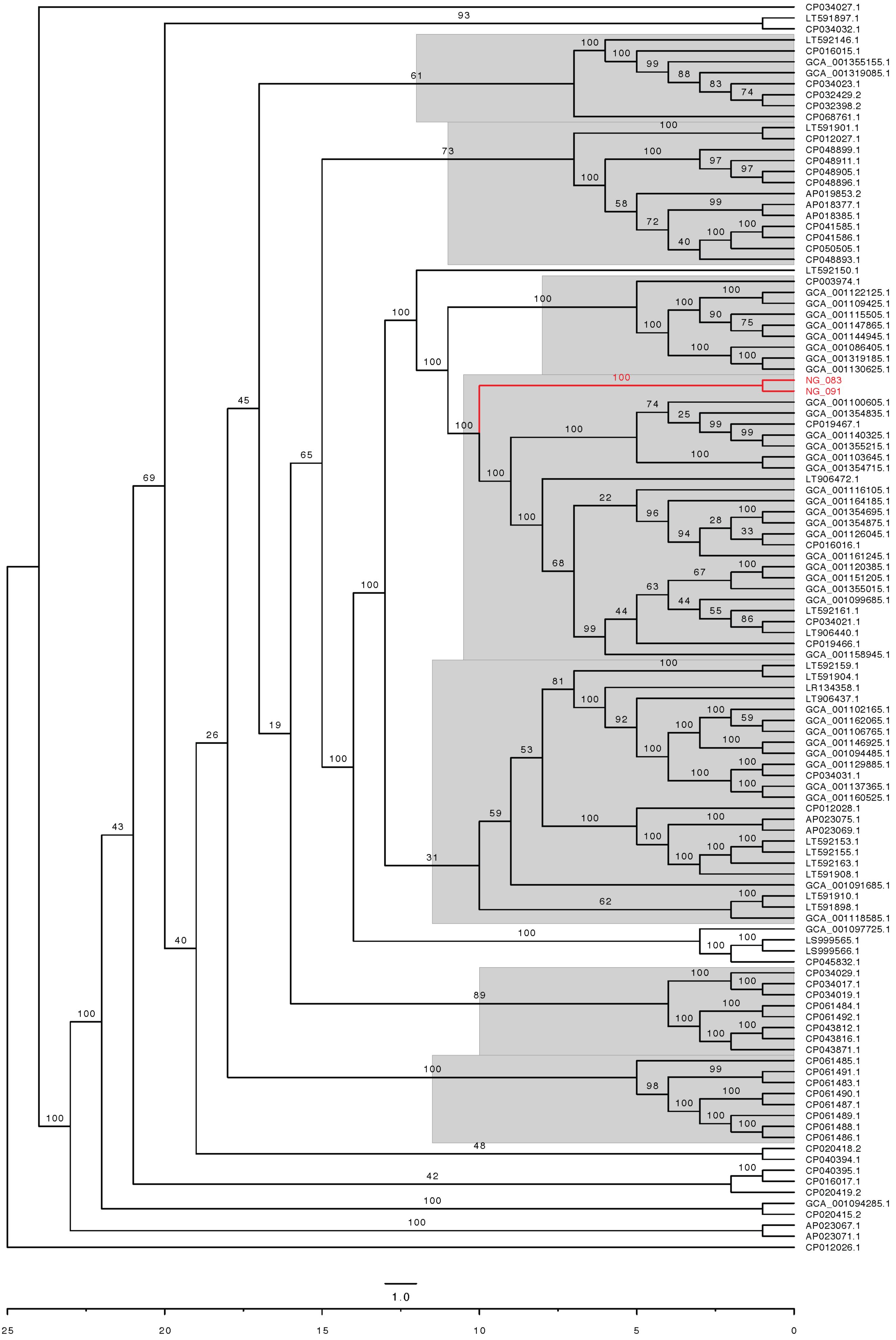

Supplement: Supplementary file 3 — Supplementary Information 3. [file 41598_2021_675_MOESM3_ESM.pdf]
