# Supplementary material for: Multidrug-resistant Neisseria gonorrhoeae infection in heterosexual men with reduced susceptibility to ceftriaxone, first report in Thailand
Source: Sci Rep. 2021 Nov 4;11:21659. doi: 10.1038/s41598-021-00675-y (PMC8569152; doi:10.1038/s41598-021-00675-y)
Supplement: Supplementary file 5 — Supplementary Information 5. [file 41598_2021_675_MOESM5_ESM.docx]

**Multidrug-resistant *Neisseria gonorrhoeae* infection in heterosexual men with reduced susceptibility to ceftriaxone, first report in Thailand**

Naris Kueakulpattana ^1,3,#^, Dhammika Leshan Wannigama^1,2,3,#^, Sirirat Luk-in^4, #^, Parichart Hongsing^5, 6, #^, Cameron Hurst^7, #^, , Vishnu Nayak Badavath^8, 9,†^, Piroon Jenjaroenpun^10,†^, Thidathip Wongsurawat^10, †^, Nipat Teeratakulpisan^11, †^, Stephen J Kerr^12,13^, Shuichi Abe^14^, Phatthranit Phattharapornjaroen ^15, †^, Aye Mya Sithu Shein^1,3^, Thammakorn Saethang^16,†^, Naphat Chantaravisoot^17,18, †^, Mohan Amarasiri^19^, Paul G. Higgins^20,21, †^ , Tanittha Chatsuwan^1,3,*^

^1^ Department of Microbiology, Faculty of Medicine, Chulalongkorn University, King Chulalongkorn Memorial Hospital, Thai Red Cross Society, Bangkok, Thailand

^2^ School of Medicine, Faculty of Health and Medical Sciences, The University of Western Australia, Nedlands, Western Australia, Australia.

^3^Antimicrobial Resistance and Stewardship Research Unit, Faculty of Medicine, Chulalongkorn University, Bangkok, Thailand.

^4^ Department of Clinical Microbiology and Applied Technology, Faculty of Medical Technology, Mahidol University, Bangkok, Thailand.

^5^ Mae Fah Luang University Hospital, Mae Fah Luang University, Chiang Rai, Thailand.

^6^ School of Integrative Medicine of Mae Fah Luang University, Chiang Rai, Thailand.

^7^ Department of Statistic, QIMR Berghofer Medical Research Institute, Brisbane, Queensland, Australia.

^8^ Institute for Drug Research, The Hebrew University, Jerusalem 9112001, Israel

^9^Chitkara College of Pharmacy, Chitkara University, Punjab, 140401, India

^10^ Division of Bioinformatics and Data Management for Research, Department of Research and Development, Faculty of Medicine, Siriraj Hospital, Mahidol University, Bangkok, 10700, Thailand

^11^ The Thai Red Cross AIDS Research Centre, Bangkok, Thailand.

^12^ HIV-NAT, Thai Red Cross AIDS Research Centre, Bangkok, Thailand.

^13^ Center of Excellence in Biostatistics, Faculty of Medicine, Chulalongkorn University, Bangkok, Thailand.

^14^ Department of Infectious Diseases and Infection Control, Yamagata Prefectural Central Hospital, Yamagata, Japan.

^15^ Department of Emergency Medicine, Center of Excellence, Faculty of Medicine Ramathibodi Hospital, Mahidol University, Bangkok, Thailand

^16^ Department of Computer Science, Faculty of Science, Kasetsart University, Bangkok, Thailand.

^17^ Office of Research Affairs, Faculty of Medicine, Chulalongkorn University, Bangkok, Thailand.

^18^ Department of Biochemistry, Faculty of Medicine, Chulalongkorn University, Bangkok, Thailand.

^19^ Laboratory of Environmental Hygiene, Department of Health Science, School of Allied Health Sciences, Kitasato University, Kitasato, Sagamihara-Minami, Kanagawa, 252-0373, Japan.

^20^ Institute for Medical Microbiology, Immunology and Hygiene, Faculty of Medicine and University Hospital Cologne, University of Cologne, Cologne, Germany.

^21^ German Centre for Infection Research, Partner site Bonn-Cologne, Cologne, Germany.

^#^ These authors also contributed equally to this work as first authors

^†^ These authors also contributed equally to this work

*Corresponding author: Tanittha Chatsuwan, PhD

**Supplementary Material and Methods**

**Phenotypic antimicrobial susceptibility testing**

The minimum inhibitory concentrations (MICs) (mg/L) of nine antimicrobials (penicillin G, tetracycline, ciprofloxacin, azithromycin, cefixime, ceftriaxone, ertapenem, gentamicin (Sigma-Aldrich (St. Louis, MO, USA) and Fosfomycin (Wako Chemicals, Tokyo, Japan) (Supplemented with 25 mg/L of glucose-6-phosphate (G-6-P) (Sigma-Aldrich, USA) were interpreted in accordance with Clinical and Laboratory Standards Institute (CLSI) clinical breakpoint guidelines. using the both broth micro-dilution (fastidious broth (FB) supplemented with 1% IsoVitaleX) and plate dilution methods (Supplementary Table 1). However, ertapenem, fosfomycin and gentamicin susceptibilities were interpreted according to the criteria for *Enterobacteriaceae* from the CLSI^1^. Azithromycin susceptibility was interpreted in accordance with EUCAST clinical breakpoint criteria. In the present study *in vitro* decreased susceptibility to ceftriaxone was defined as having an MIC of >0.064–0.125 mg/L.

**Biofilm formation, quantiﬁcation and classification**

Biofilm formation in a 96-well-microtitre-plate format was performed as described previously. Initially, a pure culture of a single colony of *N. gonorrhoeae* was inoculated into 2 mL of FB supplemented with 1% IsoVitaleX medium in a tube and incubated in an orbital shaker (200 rpm) at 37°C overnight for about 16 h. Subsequently, a subculture was prepared from the overnight culture by diluting it with fresh FB supplemented with 1% IsoVitaleX medium to an optical density (OD) of 0.02 at 600 nm (5 × 10^7^ CFU/mL) and 100 μL aliquots were added in triplicate to flat-bottomed 96-well polystyrene microtitre plates (SPL Life Sciences), with uninoculated FB in triplicate as a negative control, the plates were incubated at 37°C for 24 h.

**Phylogenetic analyses**

The core genome from draft genomes of *N. gonorrhoeae* NG_83 and NG_91 clinical isolated in Thailand and from varying *N. gonorrhoeae* WGS investigations conducted elsewhere (available on NCBI database) were determined for the number of single nucleotide polymorphisms (SNPs) by Core-Genome SNP Analysis. A reference was randomly selected among the genome sequences to generate a core genome alignment and phylogenetic tree was constructed using a core SNP alignment. Draft genomes of *N. gonorrhoeae* were aligned following the detection and filtration of recombinant regions using Parsnp v1.2 (1) and Gubbins v2.4.1 (2). Maximum-likelihood (ML) trees were generated by RAxML v8.2.12 (3) using ASC_GTRGAMMA model of rate heterogeneity with the Lewis correction for ascertainment bias (4, 5). Best-scoring ML tree was visualised and annotated as a phylogenetic tree using FigTree v1.4.4 and Evolview v2 (6, 7). The ML phylogenetic tree was built on the 21,533 core SNPs of non-recombinant core genome from 114 draft genomes from *N. gonorrhoeae* NG_83 and NG_91 clinical isolates and from a global selection of isolates (available on NCBI database). CP061489.1, from BioProject accession no. PRJNA660404, the next closest related lineage was selected as an appropriate outlying group to root the phylogenetic tree (Figure 7).

**Molecular docking**

Molecular docking studies were carried out to understand binding mode analysis and orientation of ceftriaxone in the active site of PBP2 and compared with mutative penicillin-binding protein 2 (mPBP2). Due to the lack of the crystal structure of the Ceftriaxone and mPBP2 in complex form, initially it was challenging to build 3D coordinates for subsequent computational analysis. The standard macromolecule was extracted from rcsb.org/ (**PDB: 3EQU**). The structure was visualized and the mutations were modeled by PyMOL [DeLano, 2002] (The **PyMOL** Molecular Graphics System, Version 1.2r3pre, Schrödinger, LLC.). Both the receptors were prepared in a same manner by deleting the co-crystalized waters, adding polar hydrogen and compute gasteiger charge. Even the ligand was treated by adding polar hydrogen and compute gasteiger charge, before initiating the docking process. The grid file was generated with 40 × 40 × 40 as number of points in x, y, and z directions and the center spacing were of 0.375Å. A docking output file was prepared with Lamarckian Genetic Algorithm using default settings. At the end, both the grid file and docking output files were run using script of autogrid4 and autodock4 (The Scripps Research Institute). The interactions were analyzed using MGL tools.

**Checkerboard and Time-kill assays**

The synergistic activities of antibiotics combinations including ceftriaxone plus azithromycin, ceftriaxone plus fosfomycin, ceftriaxone plus gentamicin, and ceftriaxone plus ertapenem were screened against 2 strains of *N. gonorrhoeae* isolates with reduced susceptibility to ceftriaxone using checkerboard method to determine the fractional inhibitory concentration index (FICI) according to previous method. The FICI were calculated and interpreted as described previously ^2^. Two strains of *N. gonorrhoeae* with reduced susceptibility to ceftriaxone that showed the best synergistic activity (ceftriaxone plus azithromycin) by checkerboard method was confirmed using time-kill assay. The each antibiotic at concentration 0.125X, 0.25X, and 0.5X for ceftriaxone and at concentration 0.5X and 1X MIC for azithromycin, and antibiotic combination were 0.125X MIC of ceftriaxone plus 0.5X MIC of azithromycin, 0.125X MIC of ceftriaxone plus 1X MIC of azithromycin, 0.25X MIC of ceftriaxone plus 0.5X MIC of azithromycin, and 0.25X MIC of ceftriaxone plus 1X MIC of azithromycin were incubated with 10^6^ CFU/ml *N. gonorrhoeae* isolates with reduced susceptibility to ceftriaxone. The viable bacterial cells were determined at 0, 2, 4, 6, 8, 12, and 24 h after incubation with shaking at 37 °C, 5% CO_2_. All experiments were performed at least three times. Bactericidal activity was defined as a ≥ 3log10 decrease in CFU/mL when compared to growth control curve. The synergistic activities were interpreted as follow: Synergistic: ≥ 2log_10_ (CFU/ml)-fold decrease in combination compared with single antibiotic. Antagonistic: ≥ 2log_10_ (CFU/ml)-fold increase in combination compared with single antibiotic. Bactericidal activity was defined as a ≥ 3log10 decrease in CFU/mL when compared to growth control curve. The synergistic activities were interpreted as follow: Synergistic: ≥ 2log_10_ (CFU/ml)-fold decrease in combination compared with single antibiotic. Antagonistic: ≥ 2log_10_ (CFU/ml)-fold increase in combination compared with single antibiotic.

Supplementary Table 1. Specific primers for amplification of *carA* and *orf1* genes

| Gene locus | Primer | Sequence (5’-3’) | Product size (bp) | Reference |
| --- | --- | --- | --- | --- |
| *carA* | NgCPS1 | CGGCATCGTAGCGCGCACAG | 412 | ^3^ |
|  | NgCPS4 | CGGCTGCTGTCGTCGGCGGAAT |  |  |
| *orf1* | orf1-F | CAACTATTCCCGATTGCGA | 260 | ^4^ |
|  | orf1-R | GTTATACAGCTTCGCCTGAA |  |  |

Supplementary Table 2. MIC interpretive standards (mg/L) for *N. gonorrhoeae*

| Antimicrobial agents | MIC interpretive criteria (mg/L) | | | Reference |
| --- | --- | --- | --- | --- |
|  | susceptible | intermediate | resistant |  |
| penicillin G | ≤ 0.06 | 0.12-1 | ≥ 2 | ^5^ |
| tetracycline | ≤ 0.25 | 0.5-1 | ≥ 2 |  |
| ciprofloxacin | ≤ 0.06 | 0.12-0.5 | ≥ 1 |  |
| cefixime | ≤ 0.25 | - | - |  |
| ceftriaxone | ≤ 0.25 | - | - |  |
| azithromycin | ≤ 0.25 | - | > 0.5 | ^6^ |
| gentamicin* | ≤ 4 | 8 | ≥ 16 | ^5^ |
| fosfomycin* | ≤ 64 | 128 | ≥ 256 |  |
| ertapenem* | ≤ 0.5 | 1 | ≥ 2 |  |

*Ertapenem, fosfomycin and gentamicin susceptibilities were interpreted according to criteria for *Enterobacteriaceae* from the CLSI no breakpoint is available for *N. gonorrhoeae*.

Supplementary Table 3. Specific primers for amplification and sequencing of ceftriaxone resistance mechanisms

| Gene locus (activity) | Primer | Sequence (5’-3’) | Product size (bp) | Reference |
| --- | --- | --- | --- | --- |
| *penA* (PBP2) | penA-A1  penA-B1 | CGGGCAATACCTTTATGGTGGAAC  AACCTTCCTGACCTTTGCCGTC | 668 | ^7^ |
|  | penA-A2 penA-B2 | AAAACGCCATTACCCGATGGG TAATGCCGCGCACATCCAAAG | 583 | ^7^ |
|  | penA-A3 penA-B3 | GCCGTAACCGATATGATCGA CGTTGATACTCGGATTAAGACG | 862 | ^7^ |
| *ponA* (PBP1) | PonA-F PonA-R | GAGAAAATGGGGGAGGACCG GGCTGCCGCATTGCCTGAAC | 206 | ^8^ |
| *mtrR* (MtrCDE efflux pump) | MtrR-F  MtrR-R | GCCAATCAACAGGCATTCTTA  GTTGGAACAACGCGTCAAAC | 401 | ^8^ |
| *porB* (outer membrane proteins) | PorB-F  PorB-R | CCGGCCTGCTTAAATTTCTTA  TATTAGAATTTGTGGCGCAG | 873 | ^9^ |

Supplementary Table 4. Specific primers for amplification and sequencing of two highly polymorphic antigen-encoding loci

| Gene locus | Primer | Sequence (5’-3’) | Product size (bp) | Reference |
| --- | --- | --- | --- | --- |
| *por* | Por-F  Por-R | CAAGAAGACCTCGGCAA  CCGACAACCACTTGGT | 737 | ^10^ |
| *tbpB* | TbpB-F  TbpB-R | CGTTGTCGGCAGCGCGAAAAC  TTCATCGGTGCGCTCGCCTTG | 535 | ^10^ |

Reference

1 Tesh, L. D. *et al.* Neisseria gonorrhoeae and fosfomycin: Past, present and future. *Int J Antimicrob Agents* **46**, 290-296, doi:10.1016/j.ijantimicag.2015.05.007 (2015).

2 Pereira, R., Cole, M. J. & Ison, C. A. Combination therapy for gonorrhoea: *in vitro* synergy testing. *The Journal of antimicrobial chemotherapy* **68**, 640-643, doi:10.1093/jac/dks449 (2013).

3 Mayta, H. *et al.* Use of a reliable PCR assay for the detection of *Neisseria gonorrhoeae* in Peruvian patients. *Clinical microbiology and infection* **12**, 809-812, doi:10.1111/j.1469-0691.2006.01452.x (2006).

4 Chaudhry, U. & Saluja, D. Detection of *Neisseria gonorrhoeae* by PCR using *orf1* gene as target. *Sexually transmitted infections* **78**, 72, doi:10.1136/sti.78.1.72 (2002).

5 CLSI. Performance Standards for Antimicrobial Susceptibility Testing, 31st Edition. *Clinical and Laboratory Standards Institute* (2021).

6 EUCAST. Clinical breakpoints and dosing of antibiotics *European Committee for Antimicrobial Susceptibility Testing* **v 11.0** (2021).

7 Ito, M. *et al.* Emergence and spread of *Neisseria gonorrhoeae* clinical isolates harboring mosaic-like structure of penicillin-binding protein 2 in Central Japan. *Antimicrobial agents and chemotherapy* **49**, 137-143, doi:10.1128/aac.49.1.137-143.2005 (2005).

8 Ilina, E. N. *et al.* Relation between genetic markers of drug resistance and susceptibility profile of clinical *Neisseria gonorrhoeae* strains. *Antimicrobial agents and chemotherapy* **52**, 2175-2182, doi:10.1128/AAC.01420-07 (2008).

9 Liao, M. *et al.* Clusters of circulating *Neisseria gonorrhoeae* strains and association with antimicrobial resistance in Shanghai. *The Journal of antimicrobial chemotherapy* **61**, 478-487, doi:10.1093/jac/dkm544 (2008).

10 Martin, I. M., Ison, C. A., Aanensen, D. M., Fenton, K. A. & Spratt, B. G. Rapid sequence-based identification of gonococcal transmission clusters in a large metropolitan area. *The Journal of infectious diseases* **189**, 1497-1505, doi:10.1086/383047 (2004).
