# Supplementary material for: Multidrug-resistant Neisseria gonorrhoeae infection in heterosexual men with reduced susceptibility to ceftriaxone, first report in Thailand
Source: Sci Rep. 2021 Nov 4;11:21659. doi: 10.1038/s41598-021-00675-y (PMC8569152; doi:10.1038/s41598-021-00675-y)
Supplement: Supplementary file 6 — Supplementary Information 6. [file 41598_2021_675_MOESM6_ESM.docx]

**Multidrug-resistant *Neisseria gonorrhoeae* infection in heterosexual men with reduced susceptibility to ceftriaxone, first report in Thailand**

Naris Kueakulpattana ^1,3,#^, Dhammika Leshan Wannigama^1,2,3,#^, Sirirat Luk-in^4, #^, Parichart Hongsing^5, 6, #^, Cameron Hurst^7, #^, , Vishnu Nayak Badavath^8, 9,†^, Piroon Jenjaroenpun^10,†^, Thidathip Wongsurawat^10, †^, Nipat Teeratakulpisan^11, †^, Stephen J Kerr^12,13^, Shuichi Abe^14^, Phatthranit Phattharapornjaroen ^15, †^, Aye Mya Sithu Shein^1,3^, Thammakorn Saethang^16,†^, Naphat Chantaravisoot^17,18, †^, Mohan Amarasiri^19^, Paul G. Higgins^20,21, †^ , Tanittha Chatsuwan^1,3,*^

^1^ Department of Microbiology, Faculty of Medicine, Chulalongkorn University, King Chulalongkorn Memorial Hospital, Thai Red Cross Society, Bangkok, Thailand

^2^ School of Medicine, Faculty of Health and Medical Sciences, The University of Western Australia, Nedlands, Western Australia, Australia.

^3^Antimicrobial Resistance and Stewardship Research Unit, Faculty of Medicine, Chulalongkorn University, Bangkok, Thailand.

^4^ Department of Clinical Microbiology and Applied Technology, Faculty of Medical Technology, Mahidol University, Bangkok, Thailand.

^5^ Mae Fah Luang University Hospital, Mae Fah Luang University, Chiang Rai, Thailand.

^6^ School of Integrative Medicine of Mae Fah Luang University, Chiang Rai, Thailand.

^7^ Department of Statistic, QIMR Berghofer Medical Research Institute, Brisbane, Queensland, Australia.

^8^ Institute for Drug Research, The Hebrew University, Jerusalem 9112001, Israel

^9^Chitkara College of Pharmacy, Chitkara University, Punjab, 140401, India

^10^ Division of Bioinformatics and Data Management for Research, Department of Research and Development, Faculty of Medicine, Siriraj Hospital, Mahidol University, Bangkok, 10700, Thailand

^11^ The Thai Red Cross AIDS Research Centre, Bangkok, Thailand.

^12^ HIV-NAT, Thai Red Cross AIDS Research Centre, Bangkok, Thailand.

^13^ Center of Excellence in Biostatistics, Faculty of Medicine, Chulalongkorn University, Bangkok, Thailand.

^14^ Department of Infectious Diseases and Infection Control, Yamagata Prefectural Central Hospital, Yamagata, Japan.

^15^ Department of Emergency Medicine, Center of Excellence, Faculty of Medicine Ramathibodi Hospital, Mahidol University, Bangkok, Thailand

^16^ Department of Computer Science, Faculty of Science, Kasetsart University, Bangkok, Thailand.

^17^ Office of Research Affairs, Faculty of Medicine, Chulalongkorn University, Bangkok, Thailand.

^18^ Department of Biochemistry, Faculty of Medicine, Chulalongkorn University, Bangkok, Thailand.

^19^ Laboratory of Environmental Hygiene, Department of Health Science, School of Allied Health Sciences, Kitasato University, Kitasato, Sagamihara-Minami, Kanagawa, 252-0373, Japan.

^20^ Institute for Medical Microbiology, Immunology and Hygiene, Faculty of Medicine and University Hospital Cologne, University of Cologne, Cologne, Germany.

^21^ German Centre for Infection Research, Partner site Bonn-Cologne, Cologne, Germany.

^#^ These authors also contributed equally to this work as first authors

^†^ These authors also contributed equally to this work

*Corresponding author: Tanittha Chatsuwan, PhD

**Supplementary Results**

***N. gonorrhoeae* clinical isolates display diverse antimicrobial resistance**

The susceptibility profile and antimicrobial resistance rates of all *N. gonorrhoeae* isolates are summarized in Supplementary Table 2. There is no pattern of relationship between age, sexual preferences and different antibiotic concentration were found (Supplementary Figure 1). The most prevalent antimicrobial resistance was found against ciprofloxacin, followed by tetracycline, penicillin G, gentamicin, azithromycin, and ertapenem. The most common patterns of antimicrobial resistance among the *N. gonorrhoeae* isolates were PEN^R^ TET^R^ CIP^R^ GEN^R^ and PEN^R^ TET^R^ CIP^R^ (Supplementary Table 3). The prevalence of multidrug-resistance isolates was 82.84%, whereas only 1.49% were sensitive to all antimicrobial agents tested.

**Alterations in PBP2, PBP1, PorB, MtrR, and *mtrR* promoter region were also found in a cluster of ceftriaxone-susceptible *Neisseria gonorrhoeae* isolates**

The four isolates (NG-050, NG-079, NG-090, and NG-097) with low-level ceftriaxone MIC (range 0.008-0.06 mg/L) harbor a L421P substitution in PBP1 and an adenine deletion in the 13-bp inverted repeat sequence of the *mtrR* promoter region (Supplementary Table 4). They also had the nonmosaic PBP2 patterns II, VII, XVIII, and XXXXIV. A strain GC-013 had **a** new mosaic PBP2 pattern CXVIII like the PBP2 of *N. meningitidis* strain M38900 (GenBank accession no. WP_118824975.1) (Supplementary Figure 2). NG-090 and NG-097 had D79N, T86A and H105Y substitutions in the repressor of MtrR, and G120K with A121G substitution in PorB porin. In the repressor of MtrR, NG-050 had an A39T and G45D substitution while NG-079 had G45D only. The substitution in PorB porin included G120K with A121D for NG-050 and NG-079 with G120D.

**Mutations associated with ceftriaxone resistance mechanisms were also found in a cluster of ceftriaxone-susceptible *Neisseria gonorrhoeae* isolates**

The most frequently detected mutation in ceftriaxone-susceptible *N. gonorrhoeae* isolates led to an aspartate insertion at position 345 (D345) in the non-mosaic PBP2 patterns II, VII, XIV, XIX, XVI, XVIII, and XXXXIV (Supplementary Table 4). The L421P substitution in PBP1 encoded by the *ponA* gene was found in 10 isolates. Overexpression of the MtrCDE efflux pump was associated with an adenine deletion in the 13-bp inverted repeat sequence of the *mtrR* promoter region, while amino acid alterations in the MtrR repressor were also found in nine isolates. Details of the number of isolates relevance to different substitution were summarized in supplementary table 5.

**Ceftriaxone-susceptible *Neisseria gonorrhoeae* isolates displayed known and new STs**

In addition, there were 14 known STs and 15 new STs (based on NG-MAST) among the clinical isolates (Supplementary Table 4 and 7). The most common STs were ST9208 and ST16101 (Supplementary Table 8). The most common allele numbers of *por* and *tbpB* were *por*16 and *tbpB*18, respectively. In this study, 8 novel allele numbers of *por*, 10 novel allele numbers of *tbpB*, and 15 novel STs were also found according to the NG-MAST. Different STs were related to multi drug resistance, and there was no correlation between STs and resistance phenotypes.

Supplementary Table 1. Types of specimens of the *N. gonorrhoeae* clinical isolates Thailand, 2016-2018

| Clinical specimens | *N. gonorrhoeae* (*n*=134) | | Total  (*n*=134) (%) |
| --- | --- | --- | --- |
|  | Anonymous Clinic (*n*=117) (%) | King Chulalongkorn Memorial Hospital (*n*=17) (%) |  |
| Urethral swab | 117 (100%) | 6 (35.29%) | 123 (91.79%) |
| Cervical swab | 0 (0%) | 8 (47.06%) | 8 (5.97%) |
| Blood | 0 (0%) | 2 (11.76%) | 2 (1.49%) |
| Conjunctival swab | 0 (0%) | 1 (5.88%) | 1 (0.75%) |

Supplementary Table 2. The antimicrobial susceptibility testing and antimicrobial resistance rates of the *N. gonorrhoeae* clinical isolates (n=134) Thailand, 2016-2018

| Antimicrobial agents | No. of resistant isolates (%) | MIC (mg/L) | | |
| --- | --- | --- | --- | --- |
|  |  | MIC_50_ | MIC_90_ | MIC ranges |
| Penicillin G | 98 (73.13%) | 16 | 128 | 0.25-512 |
| Tetracycline | 110 (82.09%) | 16 | 32 | 0.25-128 |
| Ciprofloxacin | 121 (90.30%) | 2 | 4 | 0.001-32 |
| Azithromycin | 18 (13.43%) | 0.06 | 1 | 0.002-4 |
| Cefixime | 0 (0%) | 0.008 | 0.015 | 0.001-0.125 |
| Ceftriaxone | 0 (0%) | 0.002 | 0.015 | 0.001-0.125 |
| Ertapenem | 2 (1.49%) | 0.015 | 0.25 | 0.002-2 |
| Fosfomycin | 0 (0%) | 16 | 32 | 4-64 |
| Gentamicin | 80 (59.70%) | 16 | 32 | 0.5-64 |

Supplementary Figure 5. PCR screening for the presence of *carA* and *orf1* genes. (A) The presence of *carA* gene: M, 100 bp plus DNA ladder; Lanes 1, Templates, *carA*-like (412 bp) of *N. gonorrhoeae* ATCC 49226; Lanes 2-6, *N. gonorrhoeae* isolates harboring the *carA*-like gene. (B), The presence of *orf1* gene: M, 100-bp plus DNA ladder; Lanes 7, Templates, *orf1*-like (280 bp) of *N. gonorrhoeae* ATCC 49226; Lanes 8-12, *N. gonorrhoeae* isolates harboring the *orf1*-like gene.

|  | M | 1 | 2 | 3 | 4 | 5 | 6 |  | M | 7 | 8 | 9 | 10 | 11 | 12 |
| --- | --- | --- | --- | --- | --- | --- | --- | --- | --- | --- | --- | --- | --- | --- | --- |
| 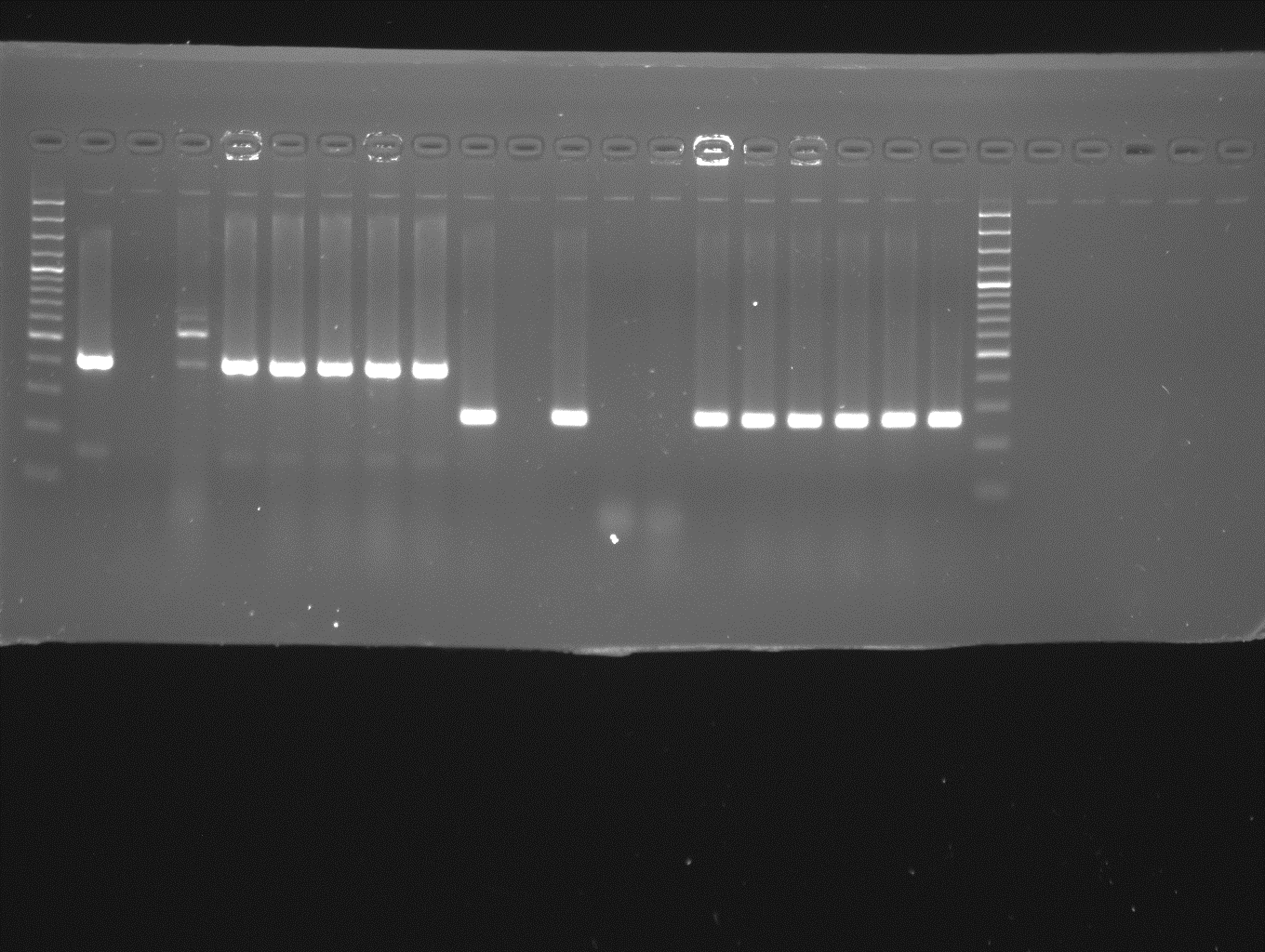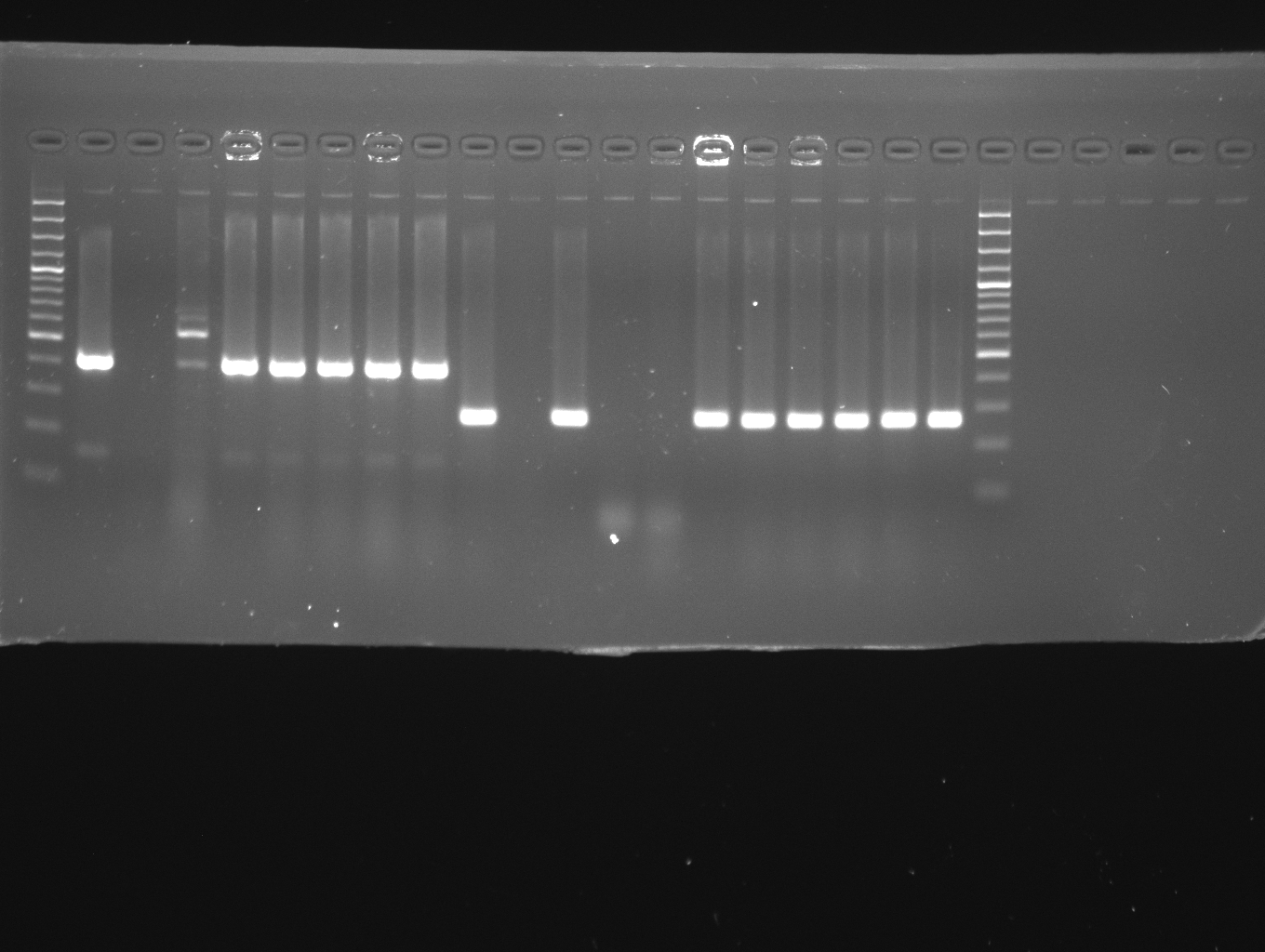 | | | | | | | | 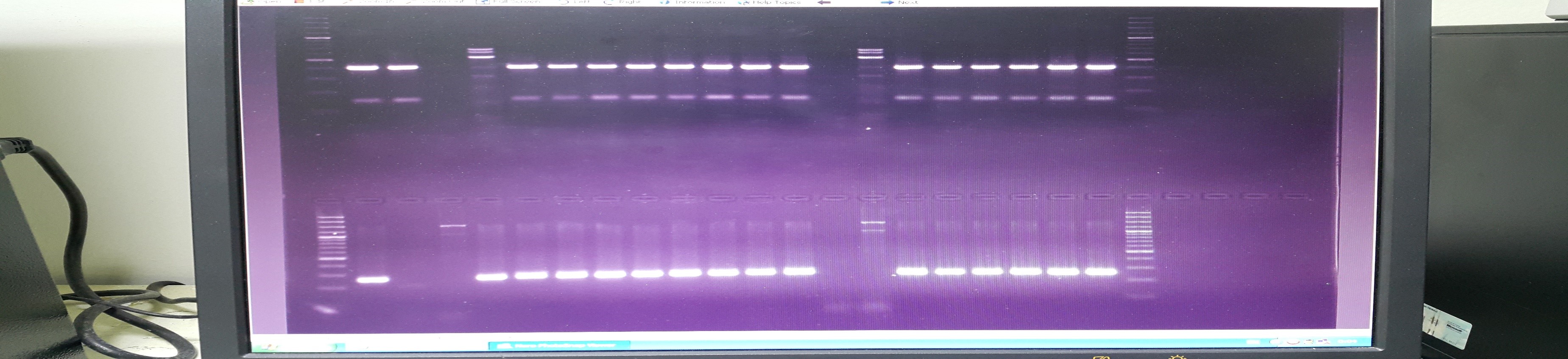 | | | | | | | |
| (A) | | | | | | | | (B) | | | | | | | |

Supplementary Table 3. Patterns of antimicrobial resistance of the *N. gonorrhoeae* (n=134) isolates Thailand, 2016-2018

| Antimicrobial agents | | No. of isolates (%) |
| --- | --- | --- |
| Resistant to 5 antibiotics (*n*=4, 2.99%) | | |
|  | PEN^R^ TET^R^ CIP^R^ AZT GEN^R^ | 4 (2.99%) |
| Resistant to 4 antibiotics (*n*=57, 42.54%) | | |
|  | PEN^R^ TET^R^ CIP^R^ GEN^R^ | 53 (39.55%) |
|  | PEN^R^ TET^R^ CIP^R^ AZT^R^ | 2 (1.49%) |
|  | PEN^R^ TET^R^ CIP^R^ ETP^R^ | 1 (0.75%) |
|  | TET^R^ CIP^R^ AZT^R^ GEN^R^ | 1 (0.75%) |
| Resistant to 3 antibiotics (*n*=50, 37.31%) | | |
|  | PEN^R^ TET^R^ CIP^R^ | 28 (20.90%) |
|  | TET^R^ CIP^R^ GEN^R^ | 8 (5.97%) |
|  | CIP^R^ AZT^R^ GEN^R^ | 4 (2.99%) |
|  | PEN^R^ CIP^R^ GEN^R^ | 3 (2.24%) |
|  | PEN^R^ TET^R^ GEN^R^ | 2 (1.49%) |
|  | TET^R^ CIP^R^ AZT^R^ | 2 (1.49%) |
|  | PEN^R^ CIP^R^ AZT^R^ | 1 (0.75%) |
|  | TET^R^ AZT^R^ GEN^R^ | 1 (0.75%) |
|  | TET^R^ AZT^R^ ETP^R^ | 1 (0.75%) |
| Resistant to 2 antibiotics (*n*=10, 7.46%) | | |
|  | TET^R^ CIP^R^ | 3 (2.24%) |
|  | CIP^R^ GEN^R^ | 3 (2.24%) |
|  | PEN^R^ TET^R^ | 2 (1.49%) |
|  | CIP^R^ AZT^R^ | 2 (1.49%) |
| Resistant to 1 antibiotic (*n*=11, 8.21%) | | |
|  | CIP^R^ | 6 (4.48%) |
|  | PEN^R^ | 2 (1.49%) |
|  | TET^R^ | 2 (1.49%) |
|  | GEN^R^ | 1 (0.75%) |
| Susceptible to all antimicrobial agents tested | | 2 (1.49%) |

Abbreviations: AZT, azithromycin; CIP, ciprofloxacin; ETP, ertapenem; GEN, gentamicin; PEN, penicillin G; TET, tetracycline

Supplementary Table 4. Mutations in resistance determinants and the β-lactam susceptibility among the *N. gonorrhoeae* clinical isolates with susceptibility to ceftriaxone (n=29), Thailand, 2016-2018

| Patient  Number | Isolate  Number | Sexual Preference | Culture Site | Penicillin MIC (mg/L) | Cefixime MIC (mg/L) | Ceftriaxone MIC (mg/L) | Resistance Determinants | | | | | NG-MAST | | |
| --- | --- | --- | --- | --- | --- | --- | --- | --- | --- | --- | --- | --- | --- | --- |
|  |  |  |  |  |  |  | PBP2 | PBP1 | *mtrR* promoter | MtrR | PorB | *por* | *tbpB* | ST |
| 18 | NG-018 | Male Heterosexual | Urethra | 512 | 0.008 | 0.008 | II | WT | WT | A39T | G120D, A121G | New allele | 1161 | New ST |
| 19 | NG-019 | Male Heterosexual | Urethra | 512 | 0.008 | 0.015 | II | WT | WT | A39T | G120K, A121N | 908 | 953 | ST8426 |
| 85 | NG-085 | Male Heterosexual | Urethra | 256 | 0.015 | 0.015 | II | WT | WT | A39T, H105Y | WT | New allele | 602 | New ST |
| 11 | GC-011 | Male Heterosexual | Urethra | 128 | 0.008 | 0.004 | II | WT | WT | A39T, H105Y | WT | New allele | New allele | New ST |
| 56 | NG-056 | Male Heterosexual | Urethra | 128 | 0.015 | 0.015 | XIX | WT | WT | A39T | WT | New allele | New allele | New ST |
| 2 | GC-002 | Female Heterosexual | Cervical | 128 | 0.015 | 0.008 | II | WT | WT | A39T, H105Y | A121G | 161 | 33 | ST16101 |
| 17 | NG-017 | Male Heterosexual | Urethra | 128 | 0.004 | 0.004 | II | WT | WT | A39T, R44H | A121S | 852 | 1412 | New ST |
| 7 | GC-007 | Female Heterosexual | Cervical | 64 | 0.03 | 0.03 | XVIII | WT | WT | A39T | WT | 8472 | 156 | ST14568 |
| 8 | GC-008 | Female Heterosexual | Cervical | 64 | 0.008 | 0.004 | II | WT | WT | A39T, H105Y | A121G | 161 | New allele | New ST |
| 12 | GC-012 | Male Heterosexual | Urethra | 64 | 0.004 | 0.004 | XIV | WT | WT | A39T | G120D, A121G | 90 | 98 | ST2485 |
| 4 | GC-004 | Male Heterosexual | Urethra | 64 | 0.015 | 0.015 | II | WT | WT | A39T | G120K, A121G | 6649 | New allele | New ST |
| 6 | GC-006 | Female Heterosexual | Cervical | 64 | 0.015 | 0.03 | II | WT | WT | A39T, H105Y | WT | New allele | New allele | New ST |
| 5 | GC-005 | Male Heterosexual | Urethra | 64 | 0.008 | 0.002 | XVI | WT | WT | A39T | WT | 30 | 2583 | ST16039 |
| 3 | GC-003 | Male Heterosexual | Urethra | 32 | 0.008 | 0.004 | II | L421P | WT | A39T | G120D, A121G | 90 | 137 | ST1691 |
| 9 | GC-009 | Male Heterosexual | Urethra | 16 | 0.008 | 0.004 | II | WT | WT | A39T, H105Y | A121G | 161 | New allele | New ST |
| 13 | GC-013 | Female Heterosexual | Cervical | 16 | 0.008 | 0.004 | new | WT | WT | A39T, F96S | A121G | 1840 | 1127 | ST5334 |
| 1 | GC-001 | Female Heterosexual | Cervical | 16 | 0.008 | 0.015 | XIX | L421P | WT | A39T | G120K, A121G | 543 | 137 | ST1612 |
| 17 | GC-017 | Men who have sex with men | Conjunctival | 8 | 0.004 | 0.004 | II | WT | WT | A39T, H105Y | G120D, A121G | New allele | New allele | New ST |
| 10 | GC-010 | Female Heterosexual | Cervical | 8 | 0.015 | 0.03 | II | WT | WT | A39T, H105Y | WT | New allele | New allele | New ST |
| 50 | NG-050 | Male Heterosexual | Urethra | 2 | 0.008 | 0.008 | II | L421P | A del | A39T, G45D | G120K, A121G | 997 | 75 | ST3047 |
| 97 | NG-097 | Male Heterosexual | Urethra | 2 | 0.03 | 0.03 | XVIII | L421P | A del | H105Y | G120K, A121D | 242 | 566 | New ST |
| 90 | NG-090 | Male Heterosexual | Urethra | 4 | 0.06 | 0.06 | XXXXIV | L421P | A del | D79N, T86A, H105Y | G120K, A121D | 4440 | 563 | ST7437 |
| 79 | NG-079 | Men who have sex with men | Urethra | 0.5 | 0.06 | 0.03 | VII | L421P | A del | G45D | G120D | 242 | 566 | New ST |
| 7 | NG-007 | Men who have sex with men | Urethra | 0.5 | 0.015 | 0.015 | II | WT | A del | A39T | WT | New allele | 18 | New ST |
| 25 | NG-025 | Men who have sex with men | Urethra | 0.5 | 0.004 | 0.004 | II | WT | WT | A39T, R44H | WT | 1808 | 893 | ST4244 |
| 51 | NG-051 | Men who have sex with men | Urethra | 0.25 | 0.001 | 0.001 | II | WT | A del | H105Y | WT | 1808 | 18 | ST9208 |
| 15 | NG-015 | Men who have sex with men | Urethra | 0.25 | 0.03 | 0.03 | II | L421P | WT | A39T, R44H | WT | 161 | 33 | ST16101 |
| 16 | NG-016 | Men who have sex with men | Urethra | 0.25 | 0.03 | 0.03 | XVIII | L421P | WT | A39T | WT | 952 | 156 | ST6284 |
| 27 | NG-027 | Men who have sex with men | Urethra | 0.25 | 0.015 | 0.015 | II | WT | A del | A39T, H105Y | WT | 1808 | 18 | ST9208 |

Abbreviations: MIC, Minimum Inhibitory Concentration; WT, Wild Type; New, New Mosaic Pattern; PBP2, penicillin binding protein 2; PBP1, penicillin binding protein 1; *mtrR*, *mtrR* promoter; MtrR, MtrR repressor; PorB, PorB porin; NG-MAST, *N. gonorrhoeae* multi-antigen sequence typing; ST, sequence type; A del, adenine deletion

Supplementary Table 5. Mutations and amino acid substitutions in beta-lactam resistance determinants and the β-lactam MIC ranges among the *N. gonorrhoeae* clinical isolates (n=31) Thailand, 2016-2018

| No. of isolates | Isolate no. | MIC (mg/L) | | | PBP2 pattern | PBP1 | *mtrR* promoter | MtrR | PorB porin |
| --- | --- | --- | --- | --- | --- | --- | --- | --- | --- |
|  |  | PEN | CFM | CRO |  |  |  |  |  |
| 4 | GC-006, GC-010, GC-011, NG-085 | 8-256 | 0.008-0.014 | 0.004-0.03 | II | WT | WT | A39T, H105Y | WT |
| 3 | GC-002, GC-008, G-009 | 16-128 | 0.008-0.015 | 0.004-0.008 | II | WT | WT | A39T, H105Y | A121G |
| 2 | NG-083, NG-091 | 2 | 0.125 | 0.125 | XXXIV | L421P | A del | H105Y | G120K, A121N |
| 1 | NG-019 | 512 | 0.008 | 0.015 | II | WT | WT | A39T | G120K, A121N |
| 1 | NG-018 | 512 | 0.008 | 0.008 | II | WT | WT | A39T | G120D, A121G |
| 1 | NG-056 | 128 | 0.015 | 0.015 | XIX | WT | WT | A39T | WT |
| 1 | NG-017 | 128 | 0.004 | 0.004 | II | WT | WT | A39T, R44H | A121S |
| 1 | GC-007 | 64 | 0.03 | 0.03 | XVIII | WT | WT | A39T | WT |
| 1 | GC-004 | 64 | 0.015 | 0.015 | II | WT | WT | A39T | G120K, A121G |
| 1 | GC-012 | 64 | 0.004 | 0.004 | XIV | WT | WT | A39T | G120D, A121G |
| 1 | GC-005 | 64 | 0.008 | 0.002 | XVI | WT | WT | A39T | WT |
| 1 | GC-003 | 32 | 0.008 | 0.004 | II | L421P | WT | A39T | G120D, A121G |
| 1 | GC-001 | 16 | 0.008 | 0.015 | XIX | L421P | WT | A39T | G120K, A121G |
| 1 | GC-013 | 16 | 0.008 | 0.004 | new | WT | WT | A39T, F96S | A121G |
| 1 | GC-017 | 8 | 0.004 | 0.004 | II | WT | WT | A39T, H105Y | G120D, A121G |
| 1 | NG-090 | 4 | 0.06 | 0.06 | XXXXIV | L421P | A del | D79N, T86A, H105Y | G120K, A121D |
| 1 | NG-097 | 2 | 0.03 | 0.03 | XVIII | L421P | A del | H105Y | G120K, A121D |
| 1 | NG-050 | 2 | 0.008 | 0.008 | II | L421P | A del | A39T, G45D | G120K, A121G |
| 1 | NG-079 | 0.5 | 0.06 | 0.03 | VII | L421P | A del | G45D | G120D |
| 1 | NG-007 | 0.5 | 0.015 | 0.015 | II | WT | A del | A39T | WT |
| 1 | NG-025 | 0.5 | 0.004 | 0.004 | II | WT | WT | A39T, R44H | WT |
| 1 | NG-015 | 0.25 | 0.03 | 0.03 | II | L421P | WT | A39T, R44H | WT |
| 1 | NG-016 | 0.25 | 0.03 | 0.03 | XVIII | L421P | WT | A39T | WT |
| 1 | NG-027 | 0.25 | 0.015 | 0.015 | II | WT | A del | A39T, H105Y | WT |
| 1 | NG-051 | 0.25 | 0.001 | 0.001 | II | WT | A del | H105Y | WT |

Abbreviations: PEN, penicillin G; CFM, cefixime; CRO, ceftriaxone; WT, wild type; new, new mosaic pattern

Supplementary Table 6. Association between mutations in resistance determinants and resistance to penicillin of *N. gonorrhoeae* clinical isolates (n=31) Thailand, 2016-2018

| Resistance determinants | | PEN^R^ isolates  *n* (%) | PEN^S^ isolates  *n* (%) | *P* value |
| --- | --- | --- | --- | --- |
| PBP2 | Mutations | 24 (%) | 7 (%) | 1 |
|  | WT | 0 (0%) | 0 (0%) |  |
| PBP1 | Mutations | 7 (%) | 3 (%) | 0.40 |
|  | WT | 17 (%) | 4 (%) |  |
| *mtrR* promoter | Mutations | 5 (%) | 4 (%) | 0.08 |
|  | WT | 19 (%) | 3 (%) |  |
| MtrR | Mutations | 24 (%) | 7(%) | 1 |
|  | WT | 0 (0%) | 0 (0%) |  |
| PorB porin | Mutations | 17 (%) | 1 (%) | 0.02* |
|  | WT | 7 (%) | 6 (%) |  |

Abbreviations: PEN^R^, resistance to penicillin (MIC of 2–512 mg/L); PEN^S^, susceptible to penicillin (MIC of 0.25–0.5 mg/L); WT, wild type.

*P* values were determined by chi-square tests. **P* value for differences in percentages of mutation in PorB porin and wild type isolates among PEN^R^ and PEN^S^ isolates.

Supplementary Table 7. MICs, and sequence types of *N. gonorrhoeae* clinical isolates (n=31) Thailand, 2016-2018

| Isolate  no. | MIC (mg/L) | | | | | | NG-MAST | | | | | |  |  |  |  |  |  |  |  |  |  |  |
| --- | --- | --- | --- | --- | --- | --- | --- | --- | --- | --- | --- | --- | --- | --- | --- | --- | --- | --- | --- | --- | --- | --- | --- |
|  | PEN | TET | CIP | AZT | CFM | CRO | FOS | GEN | ETP | *por* | *tbpB* | ST |  |  |  |  |  |  |  |  |  |  |  |
| NG-018 | 512 | 32 | 32 | 0.06 | 0.008 | 0.008 | 32 | 32 | 0.004 | New allele | 1161 | New ST |  |  |  |  |  |  |  |  |  |  |  |
| NG-019 | 512 | 4 | 8 | 0.06 | 0.008 | 0.015 | 32 | 16 | 0.015 | 908 | 953 | ST8426 |  |  |  |  |  |  |  |  |  |  |  |
| NG-085 | 256 | 16 | 1 | 0.5 | 0.015 | 0.015 | 32 | 8 | 2 | New allele | 602 | New ST |  |  |  |  |  |  |  |  |  |  |  |
| GC-002 | 128 | 32 | 1 | 0.06 | 0.015 | 0.008 | 16 | 32 | 0.015 | 161 | 33 | ST16101 |  |  |  |  |  |  |  |  |  |  |  |
| GC-011 | 128 | 64 | 1 | 0.06 | 0.008 | 0.004 | 16 | 16 | 0.015 | New allele | New allele | New ST |  |  |  |  |  |  |  |  |  |  |  |
| NG-017 | 128 | 32 | 2 | 0.25 | 0.004 | 0.004 | 16 | 32 | 0.008 | 852 | 1412 | New ST |  |  |  |  |  |  |  |  |  |  |  |
| NG-056 | 128 | 64 | 8 | 0.125 | 0.015 | 0.015 | 8 | 16 | 0.008 | New allele | New allele | New ST |  |  |  |  |  |  |  |  |  |  |  |
| GC-004 | 64 | 8 | 2 | 0.06 | 0.015 | 0.015 | 32 | 32 | 0.015 | 6649 | New allele | New ST |  |  |  |  |  |  |  |  |  |  |  |
| GC-005 | 64 | 2 | 1 | 0.03 | 0.008 | 0.002 | 16 | 16 | 0.008 | 30 | 2583 | ST16039 |  |  |  |  |  |  |  |  |  |  |  |
| GC-006 | 64 | 4 | 2 | 0.06 | 0.015 | 0.03 | 32 | 16 | 0.015 | New allele | New allele | New ST |  |  |  |  |  |  |  |  |  |  |  |
| GC-007 | 64 | 32 | 1 | 0.06 | 0.03 | 0.03 | 16 | 16 | 0.03 | 8472 | 156 | ST14568 |  |  |  |  |  |  |  |  |  |  |  |
| GC-008 | 64 | 32 | 1 | 0.06 | 0.008 | 0.004 | 16 | 16 | 0.004 | 161 | New allele | New ST |  |  |  |  |  |  |  |  |  |  |  |
| GC-012 | 64 | 32 | 1 | 0.015 | 0.004 | 0.004 | 32 | 32 | 0.004 | 90 | 98 | ST2485 |  |  |  |  |  |  |  |  |  |  |  |
| GC-003 | 32 | 32 | 2 | 0.06 | 0.008 | 0.004 | 8 | 8 | 0.008 | 90 | 137 | ST1691 |  |  |  |  |  |  |  |  |  |  |  |
| GC-001 | 16 | 2 | 1 | 0.06 | 0.008 | 0.015 | 64 | 64 | 0.004 | 543 | 137 | ST1612 |  |  |  |  |  |  |  |  |  |  |  |
| GC-009 | 16 | 4 | 1 | 0.06 | 0.008 | 0.004 | 16 | 16 | 0.004 | 161 | New allele | New ST |  |  |  |  |  |  |  |  |  |  |  |
| GC-013 | 16 | 4 | 1 | 0.06 | 0.008 | 0.004 | 16 | 8 | 0.008 | 1840 | 1127 | ST5334 |  |  |  |  |  |  |  |  |  |  |  |
| GC-010 | 8 | 1 | 0.5 | 0.03 | 0.015 | 0.03 | 16 | 8 | 0.015 | New allele | New allele | New ST |  |  |  |  |  |  |  |  |  |  |  |
| GC-017 | 8 | 2 | 0.5 | 0.004 | 0.004 | 0.004 | 8 | 8 | 0.004 | New allele | New allele | New ST |  |  |  |  |  |  |  |  |  |  |  |
| NG-090 | 4 | 4 | 8 | 0.5 | 0.06 | 0.06 | 32 | 16 | 0.125 | 4440 | 563 | ST7437 |  |  |  |  |  |  |  |  |  |  |  |
| NG-050 | 2 | 128 | 8 | 0.125 | 0.008 | 0.008 | 16 | 32 | 0.03 | 997 | 75 | ST3047 |  |  |  |  |  |  |  |  |  |  |  |
| NG-083 | 2 | 4 | 4 | 1 | 0.125 | 0.125 | 16 | 16 | 1 | 908 | 1180 | ST7235 |  |  |  |  |  |  |  |  |  |  |  |
| NG-091 | 2 | 4 | 4 | 0.5 | 0.125 | 0.125 | 32 | 16 | 1 | 1914 | New allele | New ST |  |  |  |  |  |  |  |  |  |  |  |
| NG-097 | 2 | 8 | 4 | 1 | 0.03 | 0.03 | 16 | 32 | 0.25 | 242 | 566 | New ST |  |  |  |  |  |  |  |  |  |  |  |
| NG-007 | 0.5 | 2 | 2 | 0.5 | 0.015 | 0.015 | 16 | 16 | 0.008 | New allele | 18 | New ST |  |  |  |  |  |  |  |  |  |  |  |
| NG-025 | 0.5 | 1 | 4 | 0.25 | 0.004 | 0.004 | 32 | 16 | 0.008 | 1808 | 893 | ST4244 |  |  |  |  |  |  |  |  |  |  |  |
| NG-079 | 0.5 | 4 | 8 | 1 | 0.06 | 0.03 | 4 | 8 | 0.125 | 1132 | New allele | New ST |  |  |  |  |  |  |  |  |  |  |  |
| NG-015 | 0.25 | 0.5 | 2 | 0.25 | 0.03 | 0.03 | 16 | 4 | 0.008 | 161 | 33 | ST16101 |  |  |  |  |  |  |  |  |  |  |  |
| NG-016 | 0.25 | 0.5 | 2 | 0.125 | 0.03 | 0.03 | 8 | 4 | 0.008 | 952 | 156 | ST6284 |  |  |  |  |  |  |  |  |  |  |  |
| NG-027 | 0.25 | 0.5 | 8 | 0.5 | 0.015 | 0.015 | 8 | 8 | 0.008 | 1808 | 18 | ST9208 |  |  |  |  |  |  |  |  |  |  |  |
| NG-051 | 0.25 | 2 | 4 | 0.5 | 0.001 | 0.001 | 16 | 16 | 0.015 | 1808 | 18 | ST9208 |  |  |  |  |  |  |  |  |  |  |  |

Abbreviations: PEN, penicillin G; TET, tetracycline; CIP, ciprofloxacin; AZT, azithromycin; CFX, cefixime; CRO, ceftriaxone; ETP, ertapenem; GEN, gentamicin; FOS, fosfomycin; NG-MAST, *N. gonorrhoeae* multi-antigen sequence typing; ST, sequence type

Supplementary Table 8. Sequence types by NG-MAST of *N. gonorrhoeae* clinical isolates (n=31) Thailand, 2016-2018

| NG-MAST | No. of isolates | Isolates no. |
| --- | --- | --- |
| ST9208 | 2 | NG-027, NG-051 |
| ST16101 | 2 | GC-002,NG-015 |
| ST8426 | 1 | NG-019 |
| ST7437 | 1 | NG-090 |
| ST7235 | 1 | NG-083 |
| ST6284 | 1 | NG-016 |
| ST5334 | 1 | GC-013 |
| ST4244 | 1 | NG-025 |
| ST3047 | 1 | NG-050 |
| ST2485 | 1 | GC-012 |
| ST1691 | 1 | GC-003 |
| ST1612 | 1 | GC-001 |
| ST16039 | 1 | GC-005 |
| ST14568 | 1 | GC-007 |
| New ST | 1 | GC-004 |
| New ST | 1 | GC-006 |
| New ST | 1 | GC-008 |
| New ST | 1 | GC-009 |
| New ST | 1 | GC-010 |
| New ST | 1 | GC-011 |
| New ST | 1 | GC-017 |
| New ST | 1 | NG-007 |
| New ST | 1 | NG-017 |
| New ST | 1 | NG-018 |
| New ST | 1 | NG-056 |
| New ST | 1 | NG-079 |
| New ST | 1 | NG-085 |
| New ST | 1 | NG-091 |
| New ST | 1 | NG-097 |

Abbreviations: ST, sequence type

Supplementary Table 9. MICs, mutations in resistance determinants, and sequence types of *N. gonorrhoeae* clinical isolates (n=31) Thailand, 2016-2018

| Isolates no. | MIC (mg/L) | | | | | | | | | NG-MAST |
| --- | --- | --- | --- | --- | --- | --- | --- | --- | --- | --- |
|  | PEN | CFM | CRO | ETP | TET | CIP | AZT | FOS | GEN |  |
| NG-018 | 512 | 0.008 | 0.008 | 0.004 | 32 | 32 | 0.06 | 32 | 32 | New ST |
| NG-019 | 512 | 0.008 | 0.015 | 0.015 | 4 | 8 | 0.06 | 32 | 16 | ST8426 |
| NG-085 | 256 | 0.015 | 0.015 | 2 | 16 | 1 | 0.5 | 32 | 8 | New ST |
| GC-002 | 128 | 0.015 | 0.008 | 0.015 | 32 | 1 | 0.06 | 16 | 32 | ST16101 |
| GC-011 | 128 | 0.008 | 0.004 | 0.015 | 64 | 1 | 0.06 | 16 | 16 | New ST |
| NG-017 | 128 | 0.004 | 0.004 | 0.008 | 32 | 2 | 0.25 | 16 | 32 | New ST |
| NG-056 | 128 | 0.015 | 0.015 | 0.008 | 64 | 8 | 0.125 | 8 | 16 | New ST |
| GC-004 | 64 | 0.015 | 0.015 | 0.015 | 8 | 2 | 0.06 | 32 | 32 | New ST |
| GC-005 | 64 | 0.008 | 0.002 | 0.008 | 2 | 1 | 0.03 | 16 | 16 | ST16039 |
| GC-006 | 64 | 0.015 | 0.03 | 0.015 | 4 | 2 | 0.06 | 32 | 16 | New ST |
| GC-007 | 64 | 0.03 | 0.03 | 0.03 | 32 | 1 | 0.06 | 16 | 16 | ST14568 |
| GC-008 | 64 | 0.008 | 0.004 | 0.004 | 32 | 1 | 0.06 | 16 | 16 | New ST |
| GC-012 | 64 | 0.004 | 0.004 | 0.004 | 32 | 1 | 0.015 | 32 | 32 | ST2485 |
| GC-003 | 32 | 0.008 | 0.004 | 0.008 | 32 | 2 | 0.06 | 8 | 8 | ST1691 |
| GC-001 | 16 | 0.008 | 0.015 | 0.004 | 2 | 1 | 0.06 | 64 | 64 | ST1612 |
| GC-009 | 16 | 0.008 | 0.004 | 0.004 | 4 | 1 | 0.06 | 16 | 16 | New ST |
| GC-013 | 16 | 0.008 | 0.004 | 0.008 | 4 | 1 | 0.06 | 16 | 8 | ST5334 |
| GC-010 | 8 | 0.015 | 0.03 | 0.015 | 1 | 0.5 | 0.03 | 16 | 8 | New ST |
| GC-017 | 8 | 0.004 | 0.004 | 0.004 | 2 | 0.5 | 0.004 | 8 | 8 | New ST |
| NG-090 | 4 | 0.06 | 0.06 | 0.125 | 4 | 8 | 0.5 | 32 | 16 | ST7437 |
| NG-050 | 2 | 0.008 | 0.008 | 0.03 | 128 | 8 | 0.125 | 16 | 32 | ST3047 |
| NG-083 | 2 | 0.125 | 0.125 | 1 | 4 | 4 | 1 | 16 | 16 | ST7235 |
| NG-091 | 2 | 0.125 | 0.125 | 1 | 4 | 4 | 0.5 | 32 | 16 | New ST |
| NG-097 | 2 | 0.03 | 0.03 | 0.25 | 8 | 4 | 1 | 16 | 32 | New ST |
| NG-007 | 0.5 | 0.015 | 0.015 | 0.008 | 2 | 2 | 0.5 | 16 | 16 | New ST |
| NG-025 | 0.5 | 0.004 | 0.004 | 0.008 | 1 | 4 | 0.25 | 32 | 16 | ST4244 |
| NG-079 | 0.5 | 0.06 | 0.03 | 0.125 | 4 | 8 | 1 | 4 | 8 | New ST |
| NG-015 | 0.25 | 0.03 | 0.03 | 0.008 | 0.5 | 2 | 0.25 | 16 | 4 | ST16101 |
| NG-016 | 0.25 | 0.03 | 0.03 | 0.008 | 0.5 | 2 | 0.125 | 8 | 4 | ST6284 |
| NG-027 | 0.25 | 0.015 | 0.015 | 0.008 | 0.5 | 8 | 0.5 | 8 | 8 | ST9208 |
| NG-051 | 0.25 | 0.001 | 0.001 | 0.015 | 2 | 4 | 0.5 | 16 | 16 | ST9208 |

Abbreviations: AZT, azithromycin; CIP, ciprofloxacin; CFX, cefixime; CRO, ceftriaxone; ETP, ertapenem; GEN, gentamicin; FOS, fosfomycin; PEN, penicillin G; TET, tetracycline; NG-MAST, PBP2; penicillin binding protein 2; PBP1; penicillin binding protein 1; *mtrR*, *mtrR* promoter; MtrR, MtrR repressor; PorB, PorB porin; NG-MAST, *N. gonorrhoeae* multi-antigen sequence typing; ST, sequence type; WT, wild type; A del, adenine deletion; new, new mosaic pattern.

Supplementary Table 10. Resistance patterns and sequence type *(*STs*)* of *N. gonorrhoeae* clinical isolates (n=31) Thailand, 2016-2018

| Resistance pattern | No. of ST | ST |
| --- | --- | --- |
| PEN TET CIP AZT GEN | 2 | ST7235, New ST |
| PEN TET CIP GEN | 17 | ST1612, ST2485, ST3047, ST7437, ST8426, ST16039, ST16101, ST14568, 9 New ST |
| PEN TET CIP ETP | 1 | New ST |
| PEN TET CIP | 2 | ST1691, ST5334 |
| TET CIP GEN | 2 | ST9208, New ST |
| TET CIP AZT | 1 | New ST |
| CIP GEN | 1 | ST4244 |
| PEN TET | 1 | New ST |
| CIP | 3 | ST6284, ST9208, ST16101 |
| PEN | 1 | New ST |

Abbreviations: PEN, penicillin G; TET, tetracycline; CIP, ciprofloxacin; AZT, azithromycin; ETP, ertapenem; GEN, gentamicin; ST, sequence type
